# Probability of Spring Frosts, Not Growing Degree-Days, Drives Onset of Spruce Bud Burst in Plantations at the Boreal-Temperate Forest Ecotone

**DOI:** 10.3389/fpls.2020.01031

**Published:** 2020-07-22

**Authors:** Benjamin Marquis, Yves Bergeron, Martin Simard, Francine Tremblay

**Affiliations:** ^1^ Institut de Recherche sur les Forêts, Université du Québec en Abitibi Témiscamingue, Rouyn-Noranda, QC, Canada; ^2^ Département de Sciences Biologiques, Université du Québec à Montréal, Montréal, QC, Canada; ^3^ Department of Geography, Centre for Forest Research, and Centre for Northern Studies, Laval University, Québec, QC, Canada

**Keywords:** bud phenology, growing degree-days, leaf-out, photoperiod, *Picea*, spring frost, temperature, thermal acclimation

## Abstract

Climate warming-driven early leaf-out is expected to increase forest productivity but concurrently increases leaf exposure to spring frosts, which could reduce forests' net productivity. We hypothesized that due to their damaging effect on buds, spring frosts exert a stronger control on bud phenology than do growing degree-days. We monitored bud flush phenology of three white spruce seed sources (one local seed source from the boreal mixedwood forest and two seed sources from the temperate forest), one black spruce seed source originating from the boreal mixedwood forest and four nonlocal Norway spruce seed sources in 2016 and 2017 in two plantations located on both sides of the temperate-boreal mixedwood forest ecotone in eastern Canada (Quebec). We aimed to determine inter- and intraspecies variations in bud break timing and sensitivity to air temperature and photoperiod. We expected that bud break timing for boreal species and seed sources would be better synchronized with the decrease in frost probability than for nonlocal species and seed sources. We used mixed binomial regressions and AICc model selection to determine the best environmental variables predicting each transition from one stage of bud phenology to the next. At both plantation sites, white spruce bud flush began and ended earlier compared to black and Norway spruce. Buds of all spruce species were sensitive to frost probability for early phenological stages, whereas growing degree-days controlled the remaining stages. Photoperiod sensitivity was higher for white spruce compared to black and Norway spruce and reached its maximum in the temperate forest. At intraspecies level, the two southern white spruce seed sources opened their buds earlier than the local source and were more sensitive to photoperiod, which increased their exposure to spring frosts. Onset of spruce bud flush is driven by spring frosts and photoperiod, but once started, bud phenology responds to temperature. The high photoperiod sensitivity in white spruces could counterbalance climate warming and limit future premature leaf-out, whereas the low photoperiod sensitivity in black spruce should not restrain leaf-out advancement with climate warming. Our results call for adapting the temperature-driven hypotheses of ecophysiological models predicting leaf-out to include spring frost probability.

## Introduction

The observed 1°C increase in mean global air temperature above preindustrial levels (1850–2017; Allen et al., 2018) is desynchronising tree phenology from tracking the seasonal variation in air temperature by triggering earlier spring leaf-out (Parmesan and Yohe, 2003; Piao et al., 2007; Polgar et al., 2013) and later entrance into dormancy (Jeong et al., 2011; Liu et al., 2016; Fu et al., 2017). The earlier onset and the later ending of the growing season expose frost-sensitive plant organs (buds, leaves, flowers) to more frequent spring and autumn frost events (Cannell and Smith, 1986; Liu et al., 2018; Ma et al., 2018). In fact, 43% of the Northern Hemisphere has experienced an increase of more than one growing-season frost event per year over the period 1982–2012 (Liu et al., 2018). In addition, climate warming (both mean and extremes) is not equal across seasons and latitudes (Loarie et al., 2009; Brown, 2019). Winter and spring are warming faster than summer and autumn and the frequency and intensity of extreme climatic events such as heavy rainfall and droughts are rising (Easterling et al., 2000; Stott et al., 2015; Brown, 2019). Therefore, the response of trees to climate change will likely diverge across stands or populations of the same species (D'Orangeville et al., 2018; Marchand et al., 2019), increasing the need for population-specific response to climate change. However, these responses to climate change at the population level remain largely unknown, which stresses the importance of quantifying local adaptations to better forecast the fitness of forest tree species.

At the tree level, spring frosts damage both apical and cambial meristems, as well as the leaves, thereby reducing tree growth and altering tree architecture (Clements et al., 1972; Dy and Payette, 2007; Augspurger, 2009). In contrast, summer frosts can delay the formation of the following year's foliage by damaging the newly developed primordia of evergreen trees or by limiting nutrient resorption from damaged leaves in deciduous trees (Estiarte and Peñuelas, 2015). At the ecosystem level, frost damage to leaves can decrease net primary productivity of the forests (Hufkens et al., 2012).

The frequency and intensity of spring frosts are hard to predict, given their rare occurrence and dependency upon terrain complexity (Laughlin and Kalma, 1987; Lindkvist and Lindqvist, 1997; Chung et al., 2006), where cold air masses can be trapped in topographic depressions resulting in localized frost pockets across the landscape (Dy and Payette, 2007). The strong effects of spring frosts on the fitness of temperate and boreal plant species make them important drivers of species range limits (Inouye, 2008; Kollas et al., 2014; Körner et al., 2016; Du et al., 2019). Yet spring frost effects upon spring phenology have seldom been studied (Körner et al., 2016; Lenz et al., 2016). Furthermore, these later frosts have been excluded from ecophysiological models predicting leaf-out dates, thereby favoring the use of aggregated metrics such as growing degree-days (GDDs) and photoperiod (Fuchigami and Nee, 1987; Chuine, 2000; Hänninen, 2006; Linkosalo et al., 2006). Given the high daily variation that is observed for air temperature, the more predictable nature of night/day length would make photoperiod a more reliable environmental cue for preventing early dormancy release or late dormancy entrance (Partanen et al., 1998; Cooke et al., 2012; Soolanayakanahally et al., 2013; Lang et al., 2019). However, sensitivity to photoperiod varies between species and with successional status (Caffarra and Donnelly, 2011; Basler and Körner, 2012). Therefore, disentangling the relative effects that air temperature variables (growing degree-days vs. spring frosts) and photoperiod exert on the physiological processes driving tree growth is critical to better forecast climate change effects on the productivity of temperate and boreal species.

One of the best scientific records for analyzing environmental drivers of the dormancy-growth cycle of trees is the phenological observation of bud burst in spring and bud set in autumn, since these events determine the start and the end of the growing season (Aono and Kazui, 2008; Hänninen, 2016; Tang et al., 2016). Unfortunately, the performance of many ecophysiological models that have been implemented to predict the dates when buds are expected to break is under debate because: (1) no single model provides the best fit for all tree species and (2) different models using various combinations of climate and photoperiod variables lead to similar prediction of bud break dates (Basler, 2016). Therefore, the important environmental variables triggering the physiological mechanisms of bud burst cannot be identified. This discrepancy in model projections may be the results of methodological oversimplification in the input bud phenology data or the omission of significant air temperature variables, such as spring frost occurrence. In fact, the bud break sequence must be synchronized with the decrease in frost days during spring otherwise trees would recurrently be damaged by spring frosts. However, a late bursting of the buds would decrease the growing season length, potentially decreasing growth and losing competition for space, light and nutrients to trees bursting buds earlier. Thus, the bud break sequence first follows a frost avoidance trade-off followed by a rapid bud burst that maximizes the growing season length. Consequently, the various phenological stages of the bud break process might respond to different environmental cues. However, little is known about these ecological strategies driving the bud break sequence because bud phenology is traditionally analyzed using scattered discrete field observations of buds in spring, of which, only one phenological stage (the phenological stage where buds break) is analyzed, omitting the possibility that the sensitivity of buds to environmental cues may vary within the continuous process of bud break (Hannerz, 1999; Snyder et al., 1999; Polgar and Primack, 2011).

The objectives of our study were to identify the environmental drivers at each stage of the bud break process on three spruce species and various seed sources with different bud flushing dates in two plantations that were established in the northern temperate forest and in the boreal mixedwood forest of western Quebec (Canada). In each plantation, we expected that the white spruce (*Picea glauca* [Moench] Voss) would flush its buds earlier than both the black spruce (*Picea mariana* [Mill.] B.S.P.) and the Norway spruce (*Picea abies* [L.] Karst.). At the intraspecies level, we expected that in the boreal mixedwood plantation, the southern seed sources (from the temperate forest) would open their buds earlier than the local seed source (from the boreal mixedwood forest), which would increase their exposure to spring frosts and prove to be disadvantageous to early plantation productivity during years of these occurrences. For the environmental drivers of the bud break process, we expected that as long as needles are protected by the bud scales (phenological stages 0–3), the minimum air temperature and the day length would be the main environmental drivers of transition from one stage to the next. Once needles are exposed to variation in ambient air temperature (phenological stages 4–6), subsequent bud transitions would be driven by growing degree-days. We further expected that the seed source originating from the boreal mixedwood forest would better synchronize its bud break timing with frost probability compared to nonlocal seed sources.

## Materials and Methods

### Study Site

**Figure 1:**
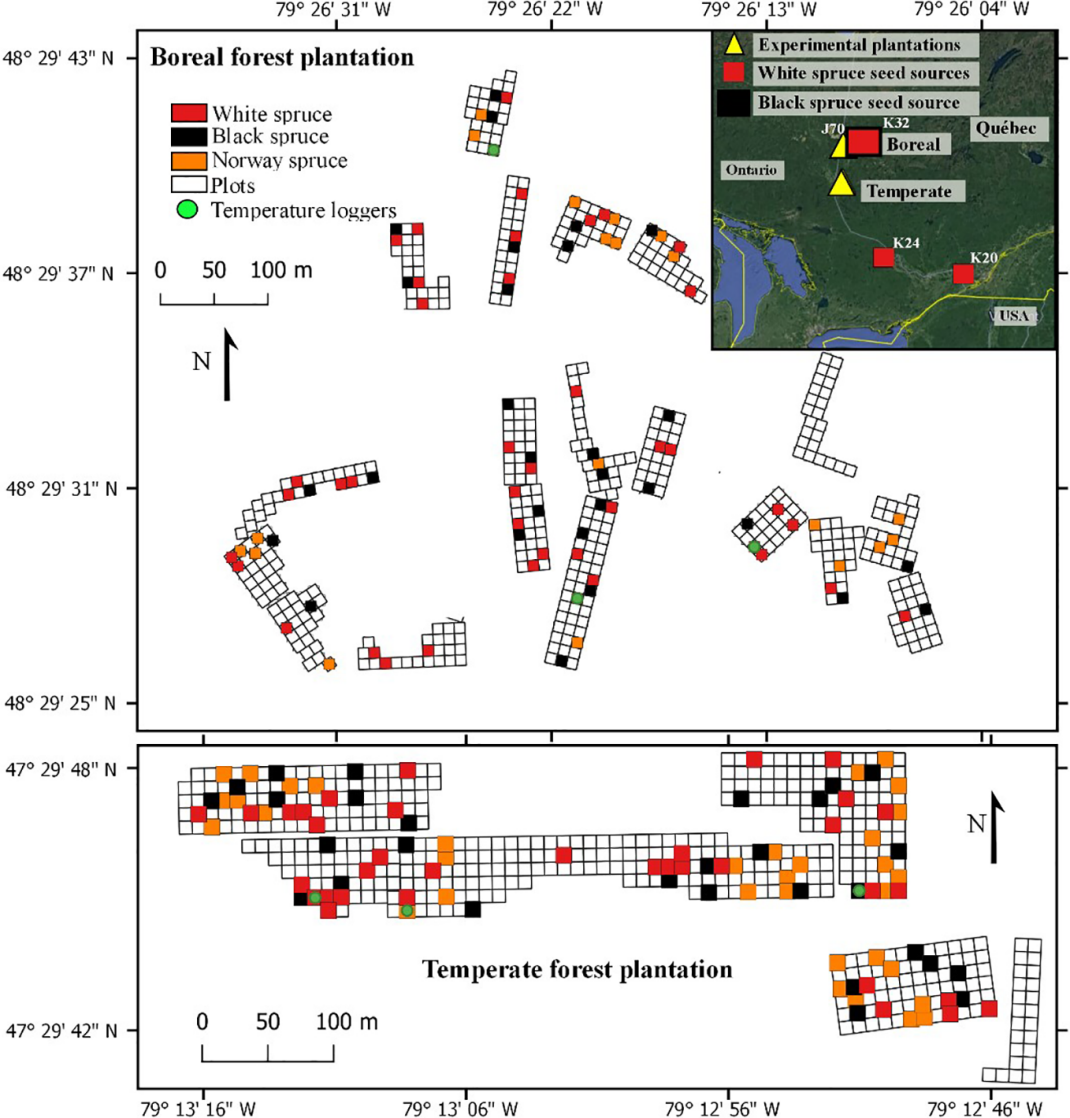
Experimental design of the plantations, the randomly sampled plots (colored squares) that were used for this study, and locations of air thermometer at the boreal mixedwood forest plantation (top) and at the temperate forest plantation (bottom). Inset shows the locations of the temperate forest and boreal mixedwood forest plantations, together with the North American seed sources.

Our study was conducted in two experimental plantations containing white spruces, black spruces, and Norway spruces. The two plantations were established in 2002; one in the northern temperate forest and one in the boreal mixedwood forest of the northern Clay Belt of Quebec (**Figure 1**). Mean annual temperature at the temperate forest plantation site (47.29° N; 79.12° W) is on average 2.1°C higher than at the boreal mixedwood forest plantation site (48.29° N; 79.26° W), with a mean annual temperature of 3.1°C and mean monthly temperatures of −15.0°C and 18.8°C in January and July, respectively (1981–2010 Normals, Barrage Angliers weather station located 24 km from the temperate forest plantation site, Environment Canada, 2019a) compared to a mean annual temperature of 1.0°C and mean monthly temperatures of −17.9°C and 16.7°C in the boreal mixedwood forest in January and July, respectively (1981–2010 Normals, Mont-Brun weather station located 47 km from the boreal mixedwood forest plantation site, Environment Canada, 2019b). At the temperate forest plantation site, the frost-free period is 46 days longer than at the boreal mixedwood forest plantation site, starting earlier by 21 days (May 24^th^, [DOY 144] vs. June 13^th^, [DOY 164]), and ending 25 days later (September 24^th^, [DOY 267] vs. August 31^st^, [DOY 243]). The last growing-season frost (0.10 probability that air temperature < 0°C) at the boreal mixedwood forest plantation site usually occurs 19 days later than in the temperate forest plantation site (June 15^th^, [DOY 166] vs. July 4^th^, [DOY 185]; 1981–2010 Normals, Barrage Angliers and Mont-Brun weather stations, Environment Canada, 2019a; Environment Canada, 2019b). These spring frosts mostly result from the temperature inversion phenomenon which generates nighttime near-ground frost events lasting few hours (Dugas, 1975; Laughlin and Kalma, 1987), which can damage both the buds and the cambium of small trees such as trees in young plantations (Dy and Payette, 2007). Precipitation is similar in the two plantation sites: the total rainfall is 709 mm vs. 705 mm and the total snowfall is 2580 vs. 2810 mm for the temperate forest and boreal mixedwood forest plantation sites, respectively. Day length in the temperate forest plantation site was eight minutes shorter during summer and eight minutes longer during winter compared to the boreal mixedwood forest plantation site (Sunset calculator, Environment Canada, 2019c).

### Experimental Design

The plantations consisted in 480 square plots of 64 m^2^ and each plot was randomly assigned to a white spruce seed sources (20 seed sources tested), a Norway spruce seed source (20 seed sources tested) or a black spruce seed source (1 seed source tested). In each plot, 25 trees of the same seed source were planted with a 2 m by 2 m spacing in five rows of five. The 20 different white spruce seed sources were collected from a first-generation seed orchard established in Cap-Tourmente in 1981 (47.06°N, 70.45°W), the 20 Norway spruce seed sources were collected from a plantation established in 1969 in Valcartier Forest Experiment Station (46.56°N, 71.30°W) for an international project on Norway spruces coordinated by the International Union of Forest Research Organizations (IUFRO) (Blouin et al., 1994; Matras, 2002). The two plantations were designed to compare the development of trees between local vs. nonlocal seed sources and to test for genetic-based adaptations and phenotypic plasticity by planting each seed sources in two contrasting climate regimes (the northern temperate forest and the boreal mixedwood forest) at the boreal-temperate forest ecotone of western Quebec (see **Figure S1** for species range maps, location of the seed sources, location of the seed orchards and the location of the plantation sites). Both local black and white spruce seed sources from the boreal mixedwood forest were used as controls. To quantify inter- and intraspecies variation in the timing of bud break, the 20 seed sources for white and Norway spruce were split into four quartiles based upon tree heights that were previously measured in 2012; one seed source per quartile was randomly selected for bud observation. For the white spruce, most trees from the lower quartile were dead at the boreal mixedwood forest plantation site. Therefore, only three seed sources (quartiles 2 to 4) were selected (see **Table 1** for the geographic locations of seed sources that were used in our study and see **Table 2** for a description of the climate at their location of origin). Only one black spruce seed source was studied because the black spruce usually flushes its buds late compared to the white spruce and, therefore, the black spruce represents a reliable benchmark for determining the impact of spring frosts on bud phenology. We avoided plots where trees were infested by the white pine weevil (*Pissodes strobi*). For the remaining plots, stratified randomly sampling was performed to select 185 plots, which were distributed across species and seed sources.

**Table 1:** Number of trees monitored, bud observations and median day of year of budbreak (phenological stage 5) per species, seed source, tree height quartile, and plantation site.

Spruce species	Seed source (height quartile)	Latitude	Longitude	Locality	Temperate forest plantation		Boreal mixedwood forest plantation	
					Number of trees (buds)	Median day of budbreak	Number of trees (buds)	Median day of budbreak
Norway	K35 (Q4)	55.15°N	30.10°E	Glubokskii, Belarus	12 (601)	165	5 (190)	164
Norway	K39 (Q3)	49.33°N	18.52°E	Istebna, Poland	13 (619)	165	2 (68)	162
Norway	K50 (Q1)	55.30°N	30.00^°^E	Gorodokskii, Belarus	15 (740)	158	4 (149)	164
Norway	K55 (Q2)	56.25°N	22.50°E	Auce, Latvia	13 (697)	155	6 (211)	155
		Total			53 (2657)		17 (618)	
White	J70 (Q4)	48.29°N	79.26°W	Duparquet, Québec	27 (1176)	155	27 (1026)	157
White	K20 (Q2)	45.36°N	74.28°W	Cushing, Québec	15 (727)	155	8 (320)	156
White	K24 (Q3)	45.54°N	77.20°W	Petawawa, Ontario	13 (557)	151	13 (247)	153
		Total			55 (2460)		48 (1593)	
Black	K32	48.29°N	79.26°W	Duparquet, Québec	51 (2357)	165	34 (1215)	164
		Grand total			159 (7474)		99 (3426)	

**Table 2:** Climate normals (1981-2010) at the location of origin of each nonlocal seed source.

Seed source (locality)	Weather station (distance to locality)	Mean annual temp. in °C	Mean January temp. (min; max) in °C	Mean July temp. (min; max) in °C	Precipitation sum in mm	Growing degree-days (base temp. 0°C)	Date with 0.10 probability of frost in spring (DOY)
K35^1^ (Glubokskii)	Smolensk (130 km)	5.5	−6.2 (−33; 9)	17.8 (5; 35)	738	2.713	May 11^th^ (131)
K39^1^ (Istebna)	Bielsko-Biala (33 km)	8.5	−1.3 (−27; 15)	18.0 (4; 34)	944	3.365	May 5^th^ (125)
K50^1^ (Gorodokskii)	Velikie-Luki (98 km)	5.8	−5.5 (−37; 11)	18.0 (3; 35)	639	2.774	May 19^th^ (139)
K55^1^ (Auce)	Siauliai (62 km)	6.8	−2.8 (−32;11)	17.8 (6; 35)	614	2.904	May 17^th^ (137)
K20^2^ (Cushing)	Lachute (12 km)	6.0	−10.7 (−37; 11)	20.4 (4; 35)	1.151	3.177	May 27^th^ (147)
K24^2^ (Petawawa)	Sheenboro (10 km)	5.0	−12.1 (−39; 11)	19.3 (1; 40)	853	2.972	June 10^th^ (161)

1 Climate data for European seed sources were retrieved from the KNMI Climate Explorer (European Climate Assessment & Data) https://climexp.knmi.nl/start.cgi.

2 Climate data for Canadian seed sources were retrieved from Environment Canada (Climate normals) https://climate.weather.gc.ca/climate_normals/.

### Bud Phenology Observations

Each bud observation was classified into one of the seven bud break stages (0–6). The stages were described by Dhont et al. (2010) for white spruce buds, by Numainville and Desponts (2004) for black spruce buds, and by Sutinen et al. (2012) for Norway spruce buds. To maintain the same number of stages per species, we added one last stage to Norway spruce, *i.e*., needles elongating and expanding laterally (**Table 3**). Buds were observed weekly from May 10^th^ until leaf-out (see **Figure S2** for the precise sampling dates). This sampling time interval was based upon previous studies analyzing bud phenology (Clark et al., 2014a; Perrin et al., 2017) and because of logistical constraints (large distance between both plantation sites) that prevented observing bud phenology at a shorter time interval. The average time delay between bud observations at one plantation and bud observations at the other plantation was of only two to three days. Even if our sampling time interval could have led to missing observations for given phenology stages, the large amount of observations collected over 258 trees increases the robustness of our data and analyses compared to more frequent bud observations collected over a limited number of individuals. The apical bud could not be observed since trees were too tall. Therefore, the terminal buds of all branches belonging to the whorl at breast height (from two to six buds per tree) were followed for two consecutive years (2016–2017). In total, 10 900 buds were observed over 258 trees (**Table 1**). Since stages one and two closely resemble one another, they were difficult to identify in the field. Therefore, data were merged for these two stages.

**Table 3:** Development stages of the spring bud phenology of white spruce (Dhont et al., 2010, p. 9), black spruce (Numainville and Desponts, 2004, pp. 10–16, Figs 6B, 7B, 8B, 9B, 10B, 11A, 12A.) and Norway spruce (Sutinen et al., 2012, p. 990, **Figure 2**).

Stages	Description	White spruce	Black spruce	Norway spruce
0	Buds are closed and dormant.	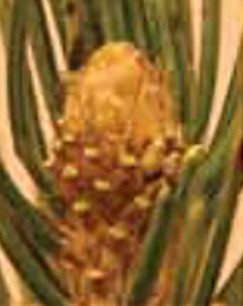	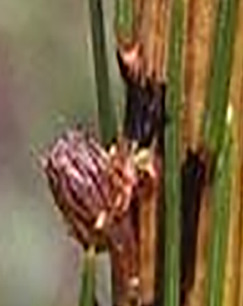	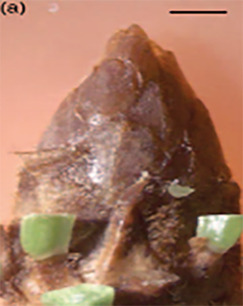
1	Bud scales are opening, and from an apical view, a white spot is visible at the top of the bud.	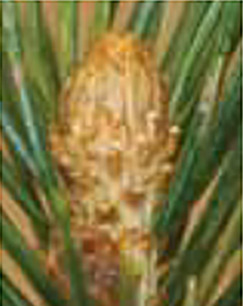	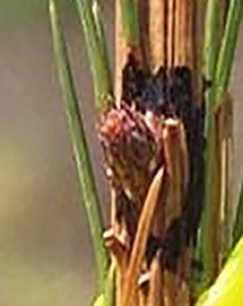	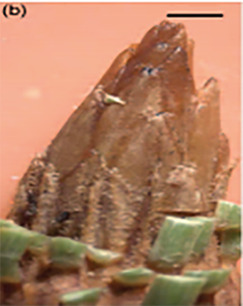
2	Buds are elongating.	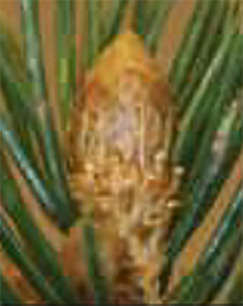	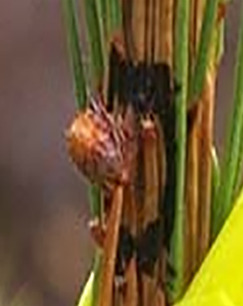	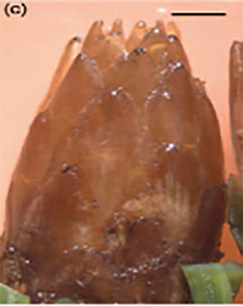
3	Buds are swelling.	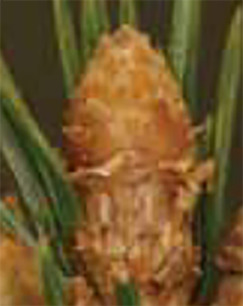	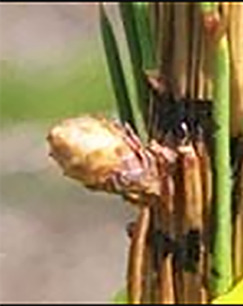	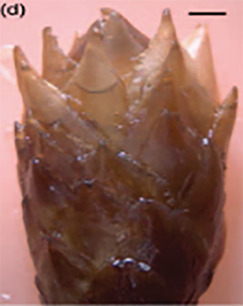
4	Bud scales are translucent, and the needles are partly visible.	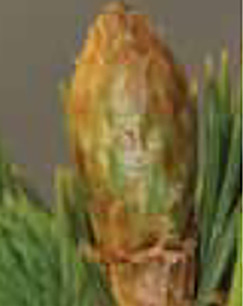	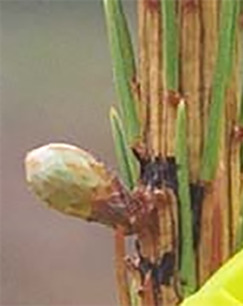	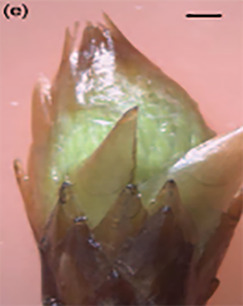
5	Bud scales are ripped at the base of the bud (white and black spruce) or open at the top (Norway spruce), and needles are tightly bundled.	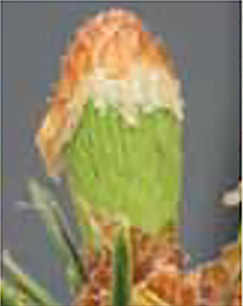	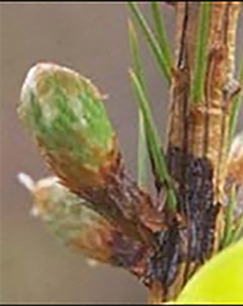	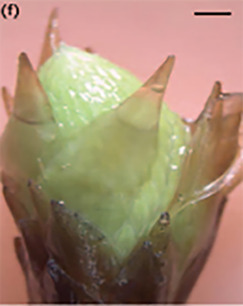
6	Needles are elongating and expanding laterally.	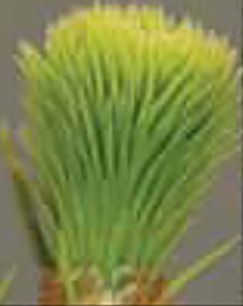	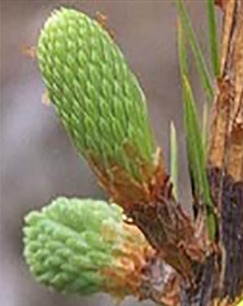	

No photograph showing buds of the Norway spruce in stage six was available.

### Climate Variables Used to Predict Timing of Bud Break

**Figure 2:**
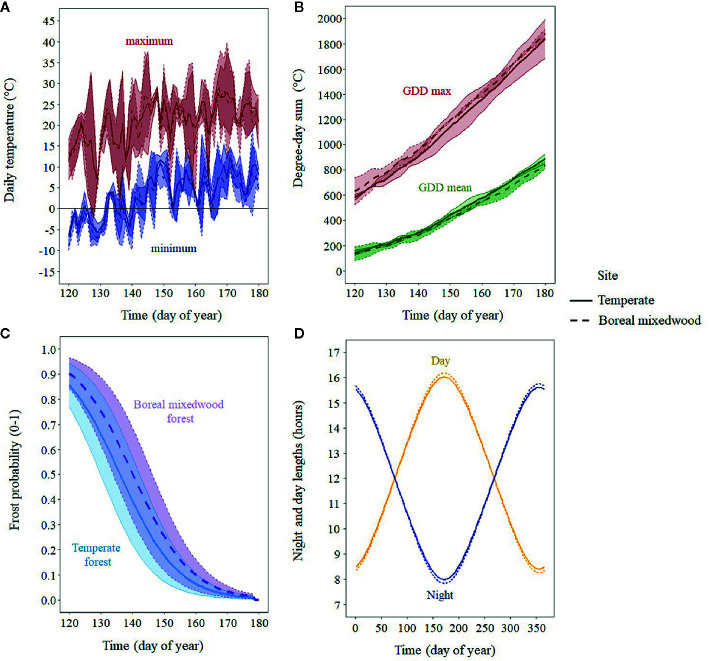
Mean (line) and standard error (shaded area) for the period 2016–2017 for air temperature and photoperiod variables that were tested: **(A)** mean daily minimum and mean daily maximum temperature; **(B)** sum of growing degree-days calculated with mean and maximum temperature; **(C)** probability of a frost event per site; and **(D)** night and day length in hours.

To analyze the timing of bud break in relation to *in situ* air temperature, we installed three thermometers (iButton DS1922L, measurement accuracy of ± 0.5°C, Maxim Integrated, San Jose, CA, USA) per plantation site set to record air temperature every 30 min from snow melt in the spring to August for two consecutive years (2016–2017). We averaged the data from the three thermometers to create one daily temperature record per site. Due to the presence of snow on the ground at the time of the first bud observations, thermometers were set 13 days later, once snow had melted. The missing air temperature data from onsite measurements were supplemented using the following approach: we used the BioSIM 10 software that was developed by Natural Resources Canada (Régnière et al., 2014) to simulate daily air temperature by accounting for elevation and aspect at both plantation sites for the entire growing season. For each plantation and temperature logger, we then regressed the air temperature that was measured in the field against the simulated air temperature values. All linear regressions were significant (*P*-values < 0.05). The mean adjusted *R^2^* of all linear regressions was 0.86, being higher for mean temperature (*R* ^2^ = 0.92) and lower for minimum (*R* ^2^ = 0.82) and maximum temperature (*R* ^2^ = 0.85; **Tables S1** and **S2**). Using the regression coefficients, we predicted the minimum, mean and maximum daily air temperature for the 13 missing days per data logger. We then averaged these predicted values per plantation and incorporated them into our observed air temperature dataset (**Figure 2A**). To determine which temperature variable best predicted each phase of the bud break process, we tested the minimum, mean and maximum daily air temperature, the sum of growing degree-days above 0°C, starting January 1^st^, which were calculated with the maximum daily air temperature (GDD max), and the mean daily air temperature (GDD mean; **Figure 2B**). We also analyzed the daily probability of occurrence of a frost event, which was calculated from the binomial regression analysis of the frequency of frost events. Days where minimum daily air temperature was below 0°C were coded 1 and the others, 0 (**Figure 2C**). We also analyzed the impact of photoperiod on bud phenology by retrieving the day length (hours of illumination during the day) from the sunset calculator (Environment Canada, 2019c) for both plantation sites (**Figure 2D**).

### Statistical Methods

**Figure 3:**
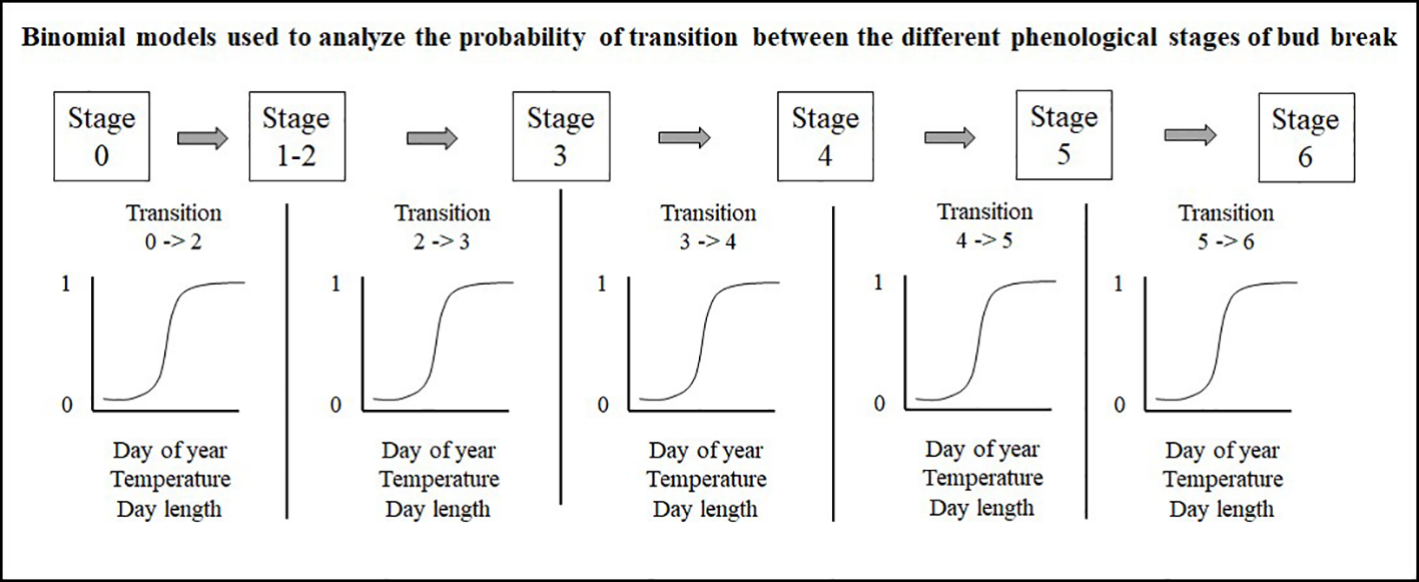
Mixed binomial regression models analyzing the probability of transition between the different phenological stages of bud break.

Prior to all analyses, numerical variables were standardized to allow comparison of the strength of coefficients from different variables. Bud observations consist in a series of stages linked by time since buds can only transit from one stage to the next. Therefore, we separately analyzed the five transitions (0– > 2; 2– > 3; 3– > 4; 4– > 5; 5– > 6) with a binomial model, using 0s for buds that remained in their current stage and 1s for buds that were transitioning to the next stage, a method also used by Perrin et al. (2017) (**Figure 3**). The site, species or seed sources, and their interactions were analyzed as fixed effect terms, whereas years and tree identity were incorporated into the random structure of the binomial regression model. We implemented these models in R using the glmer function with the bobiqa optimiser algorithm from the lme4 package (Bates et al., 2015). We considered that a bud would transit to the next stage of bud phenology once the transition probability was 0.51. We quantified bud break duration by subtracting the day of year where the transition probability of reaching stage six in the bud phenology was 0.51 from the day of the year where the transition probability of reaching combined stages one and two was also 0.51. We acknowledge that by merging stages one and two, we had underestimated the duration of the bud break process.

To determine which air temperature and photoperiod variables best predicted each phenological transition per species, we defined 31 air temperature models (**Table S3**) and six photoperiod models (**Table S4**), including a null model testing the occurrence of bud flush according to time only. This last model included day-of-year (DOY) as the predictor variable. We compared candidate models against the null using model selection, which evaluated model fit with the corrected Akaike's information criteria (AICc) (R package AICcmodavg; Mazerolle, 2017). If model selection failed to identify one best model, we used multi-model inference to average the coefficients in each probable model using the model.avg function from the R package MuMIn (Barton, 2018). To limit multicollinearity problems because of correlated climate variables and high (>10) variance inflation factors when two environmental variables co-occurred in a same model, we only tested models with single climate variables in various combinations with species, sites, and their two-way interactions. Since photoperiod and air temperature can interact to affect bud break timing (Rossi and Isabel, 2016), we first performed model selection to identify the best air temperature model per transition. We performed a second model selection to identify the best photoperiod model per transition. We then compared the best air temperature model to the best photoperiod model by analyzing the strength of their regression coefficients. Since statisticians still debate on how to properly calculate statistical significance of coefficients when regression analyses include a random structure, we considered a variable to be statistically significant if the error around the coefficient did not include zero. Once the best climate variables were identified per stage of bud phenology, we analyzed intraspecies variation in bud phenology by adding the seed source.

We restricted our analyses to the observed sequence of bud flush in spring only instead of using a dynamic model considering the interplay between spring warming and chilling requirement in autumn to model the dehardening process because 1) values for the chilling requirement were not available at the seed source level; 2) a previous study showed that the chilling requirement for spruce species were low (300–500 chilling hours) compared to other boreal and temperate tree species (trembling aspen requires 1100 chilling hours) and were completed by the end of December (Man et al., 2017), therefore unlikely impacting bud break timing in spring; and 3) we gathered bud flush data for only two years. For the latter, the possibility that warmer autumn decreases the number of accumulated chilling units in autumn, thus postponing chilling completion to early spring and consequently delaying bud break is limited compared to long-term bud phenology studies using dynamic models. Hence, the importance of chilling temperatures in controlling bud flush phenology in the spring was less of a concern in our study.

To show how our bud phenology data compared with the more conventional bud phenology analyses, we compared the sum of growing degree-days above 0°C that was required for buds to open (phenological stage five; **Table 3**) per species and site with previously published results (Snyder et al., 1999; Man and Lu, 2010).

## Results

### Inter- and Intraspecies Variation in the Timing of Bud Break

**Figure 4:**
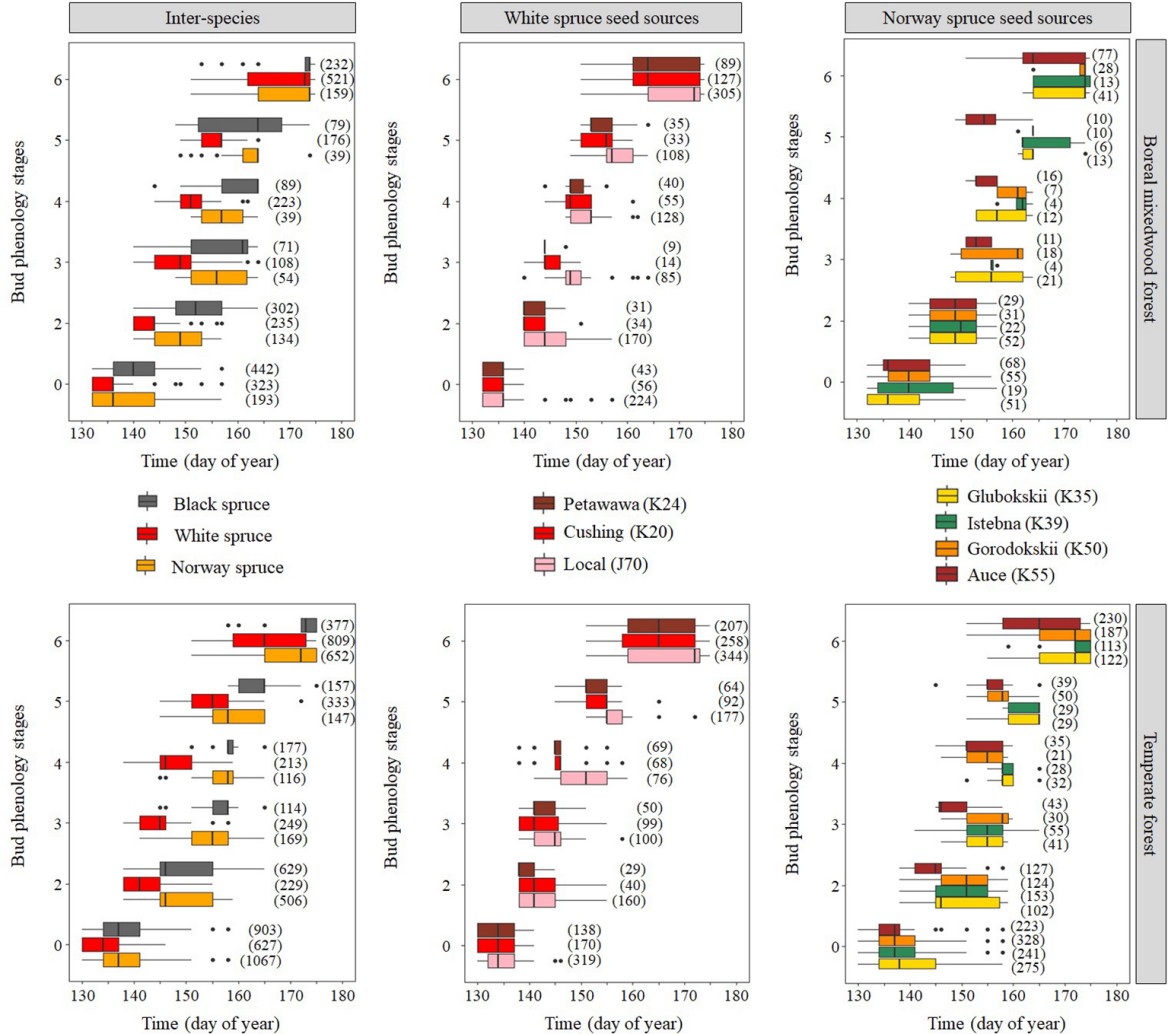
Observed distribution (box plots) of the phenological stages with time showing both inter- and intraspecies variations in the bud break process. The boxes show the 25^th^ and 75^th^ percentiles, and the line inside each box represent the median. The number in parenthesis shows the number of observations per phenological stage, species, and site.

**Figure 5:**
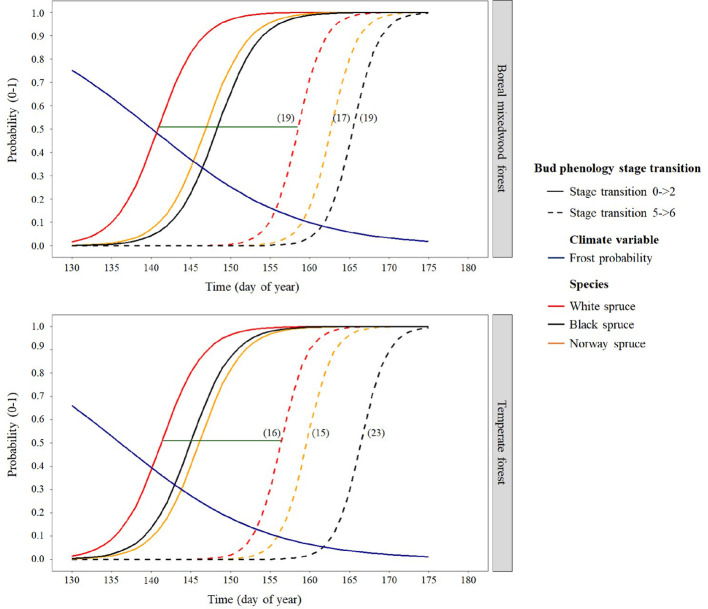
Interspecies variation in bud break timing showing the transition probabilities between the first and last transition stages of bud phenology per species, plantation site, and frost probability with time. The horizontal green line shows the duration of the bud break process for white spruce and the numbers in parentheses show the duration (in days) of the bud break process per species and site.

Results for the timing of bud break are based upon forecasts from the mixed binomial regression models predicting on which day-of-year (DOY) the transition probability was 0.51. For the white spruce, the black spruce and the Norway spruce, bud break in the temperate forest plantation was completed in 16, 23, and 15 days, respectively, compared to 19, 19, and 17 days in the boreal mixedwood forest plantation (**Figures 4** and **5**). The black spruce was the only species for which bud break phenology was faster in the boreal mixedwood forest than in the temperate forest. At both plantation sites, the white spruce reached each bud phenology stage the earliest. Also, at both plantation sites, interspecies variations were the highest for the bud swelling stage (stage three, see **Table 3**). For instance, the white spruce was the earliest species to swell buds (when the frost probability was of 0.31 and 0.33 at the temperate forest and at the boreal forest respectively) whereas both the black spruce and the Norway spruce swell buds when the frost probability was 20% and 23% lower at the temperate forest and the boreal forest respectively (**Figure 5**).

**Figure 6:**
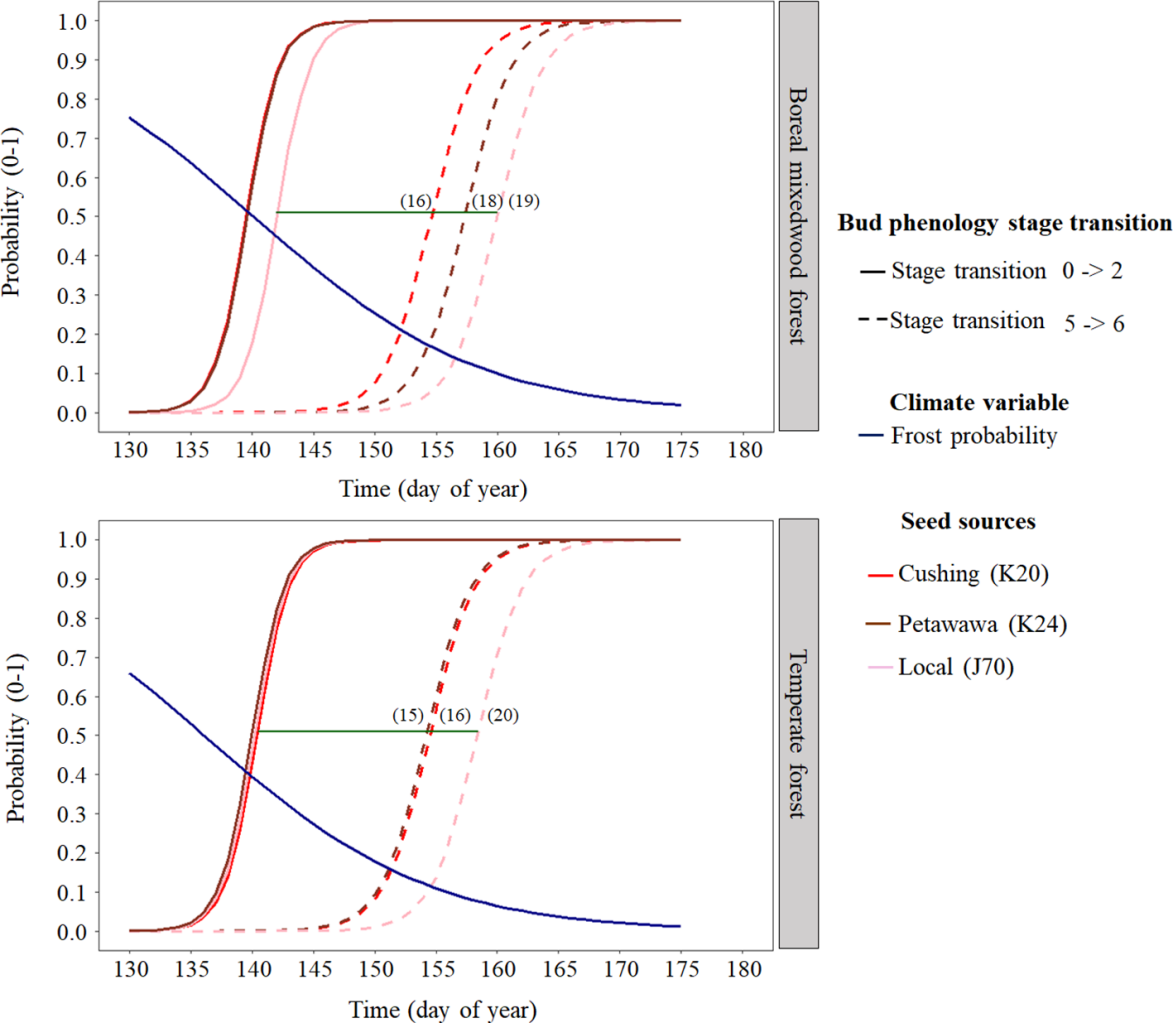
White spruce intraspecies variation in bud break timing showing the transition probabilities between the first and last transition stages of bud phenology per seed source, plantation site, and frost probability with time. The horizontal green line shows the duration of the bud break process for the local white spruce seed source naturally growing close to the boreal mixedwood forest plantation (J70) and the numbers in parenthesis show the duration (in days) of the bud break process per seed source and site.

At the intraspecies level, the nonlocal southern white spruce seed sources K20 and K24 reached each phenological stage of the bud break process earlier than the local seed source J70 (**Figures 4** and **6** and **Table 5**). In the temperate forest, white spruce intraspecies variations were the highest for the bud swelling stage (stage four). For instance, exposure to spring frost was 12% higher (from a probability of 0.41 to 0.29) for the southern seed sources compared to the local seed source originating from the boreal mixedwood forest. In the boreal mixedwood forest, white spruce intraspecies variations were highest for the translucent bud scales stage. For instance, exposure to spring frost was 10% higher (from a probability of 0.33 to 0.23) for the southern seed sources compared to the local seed source originating from the boreal mixedwood forest, which flushed its buds the latest (**Table 5**).

**Figure 7:**
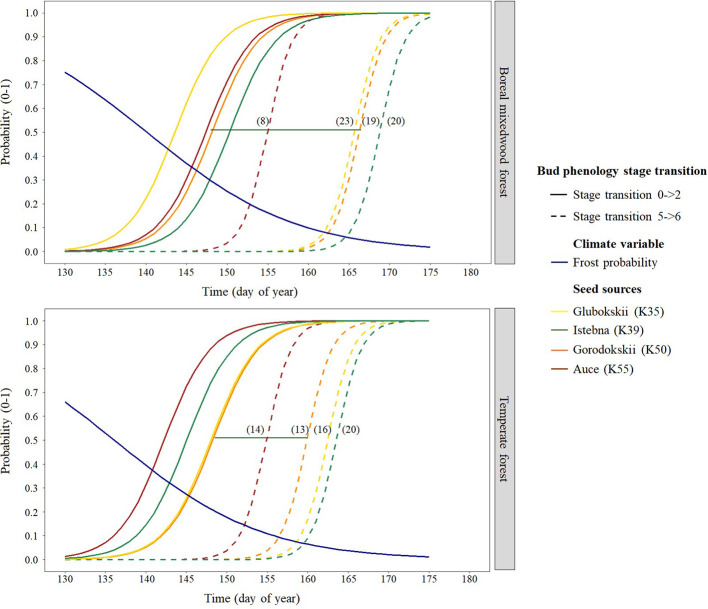
Norway spruce intraspecies variation in bud break timing showing the transition probabilities between the first and last transition stages of bud phenology per seed source, plantation site, and frost probability with time. The horizontal green line shows the duration of the bud break process for the K50 seed source naturally growing in Gorodokskii (Belarus) and the numbers in parenthesis show the duration (in days) of the bud break process per seed source and site.

For the Norway spruce, at both sites, the first and the last seed source to complete bud flush were the K55 and K39 respectively. Important differences in bud break timing were observed for intermediate bud phenology stages. Bud swelling occurred on average 7 days and 12 days earlier for the K55 seed source in the temperate forest and in the boreal mixedwood forest respectively when compared to the K39 seed source. These differences in timing of bud break increased exposure to spring frost by 7% (from a probability of 0.16 to 0.09) in the temperate forest and by 21% (from a probability of 0.33 to 0.12) in the boreal mixedwood forest (**Figures 4** and **7** and **Table 6**).

### Interspecies Variation in the Effects of Air Temperature and Photoperiod for Predicting Bud Break Phenology

**Figure 8:**
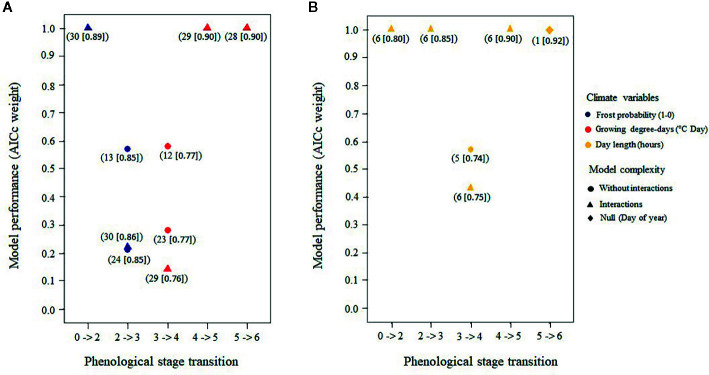
Best air temperature models **(A)** and best photoperiod models **(B)** predicting the transition of buds from a given stage to the next based upon corrected Akaike's information criteria (AICc) weights; only models with an AICc > 0.05 for each of the five bud phenology transitions are shown. Numbers in parentheses under each model refer to the candidate models prior to selection (**Tables S3** and **S4**) and the numbers in brackets show the total amount of explained variance.

Model selection on the 31 air temperature candidate models produced from one to three statistically plausible models (5%) for each stage transition (**Figure 8** and **Tables S5–S9** for complete results of the AICc model selection per stage transition). The average marginal pseudo-*R^2^* was 0.47 ± 0.15 and the conditional pseudo-*R^2^* was 0.84 ± 0.06 for the selected models. Here, the marginal value of *R* ^2^ represents the variance that was explained by the fixed effects, while the conditional value represents the variance that was explained by both fixed and random effects (Barton, 2018). The null model for bud flush solely as a function of time was always rejected (**Tables S5** to **S9**). Phenological stages where needles were still protected by the bud scales [bud elongation to bud swelling (stages two and three), see **Table 3**] were best predicted by the probability of spring frost occurrence whereas growing degree-days best predicted dates that buds reached phenological stages where needles were exposed to air temperature (stages four to six) (**Figure 8**).

Sensitivity to air temperature (both spring frost occurrence and GDDs) varied among species and sites (**Table 4** and **Tables S10–S14** for the coefficients of each climate variable per stage transition). Specifically, the white spruce was more sensitive to both the probability of spring frost occurrence and the growing degree-days compared to either the black spruce or the Norway spruce. The probability of spring frost occurrence was more important for predicting transition probabilities of buds in the boreal mixedwood compared to the temperate forest. In contrast, growing degree-days were more important for predicting transition probabilities of buds in the temperate forest plantation, particularly for the transition from phenological stage four to five (**Table 4** and **Tables S10–S14**).

**Table 4:** Results of the generalized mixed binomial regressions for inter-species variation in the timing of the bud break process.

Bud break	Frost probability (0–1)						Growing degree-days (°C, base temperature = 0°C)						Photoperiod (day length in hours)					
	Temperate forest			Boreal mixedwood forest			Temperate forest			Boreal mixedwood forest			Temperate forest			Boreal mixedwood forest		
	White spruce	Black spruce	Norway spruce	White spruce	Black spruce	Norway spruce	White spruce	Black spruce	Norway spruce	White spruce	Black spruce	Norway spruce	White spruce	Black spruce	Norway spruce	White spruce	Black spruce	Norway spruce
0 –> 2	**0.39 (140)**	0.28 (144)	0.28 (144)	**0.46 (141)**	0.35 (145)	0.34 (147)							**15.37 (140)**	15.53 (145)	**15.55 (145)**	**15.48 (141)**	15.77 (148)	**15.77 (148)**
2 –> 3	**0.31 (144)**	**0.09 (156)**	**0.13 (152)**	**0.33 (147)**	**0.08 (161)**	**0.12 (158)**	327 (143)	539 (157)	501 (155)	386 (149)	534 (160)	505 (157)	**15.40 (141)**	**15.90 (159)**	**15.81 (154)**	**15.76 (148)**	**16.04 (159)**	**15.98 (156)**
3 –> 4							**387 (147)**	**530 (157)**	**521 (156)**	397 (149)	538 (160)	532 (160)	**15.59 (147)**	**15.86 (157)**	**15.84 (156)**	**15.78 (149)**	**16.02 (158)**	**16.04 (159)**
4 –> 5							**439 (150)**	**589 (160)**	**528 (157)**	**461 (154)**	**662 (169)**	**616 (166)**	**15.70 (151)**	**15.93 (160)**	**15.85 (157)**	**15.93 (154)**	**16.11 (163)**	**16.10 (162)**
5 –> 6							**525 (156)**	**667 (167)**	**569 (160)**	523 (159)	662 (169)	565 (162)						
								Not better than the null model										
								Better than the null model but not the best model from the AICc model selection										
								Best model from AICc model selection										

The predicted value per environmental variable is shown at which the probability that a bud transitions to the next stage is 0.51 per species, while numbers in parentheses show day of year at which the transition is predicted to occur. Boldface type indicates coefficients that differ from zero, while colors indicate model performance evaluated based upon corrected Akaike's information criteria (AICc).

Model selection on the six candidate photoperiod models produced from one to two statistically plausible models (5%) for each stage transition (**Figure 8** and **Tables S15–S19** for complete results of the AICc model selection per transition stage). The average marginal pseudo-*R^2^* (i.e., fixed effects) was 0.50 ± 0.09, while the conditional pseudo-*R^2^* (i.e., fixed plus random effects) was 0.83 ± 0.08 for the selected models. The null model that tested the occurrence of bud flush solely as a function of time was rejected for the first four transitions but was the most probable model for predicting the last transition (**Figure 8** and **Tables S15–S19**). Thus, sensitivity to photoperiod was important for the first and intermediate phenological stages only. Sensitivity to photoperiod was the highest for the white spruce, followed by the Norway spruce and by the black spruce, and was higher in the temperate plantation compared to the boreal mixedwood plantation (**Table 4**, **Tables S10–S14**).

The probability of spring frost occurrence was slightly more important in predicting bud elongation (stage 2) compared to photoperiod (**Table S10**). Growing degree-days and photoperiod had a similar importance in terms of predicting intermediate phenological stages (bud swelling and translucent bud scales), whereas growing degree-days were more important than photoperiod for predicting the completion of the bud break process (**Tables S10** to **S14**). Overall, the white spruce required shorter day lengths, fewer growing degree-days, and opened its buds under higher frost probabilities than did either the black spruce or the Norway spruce (**Table 4**).

### Intraspecies Variations in the Effect of Air Temperature and Photoperiod for Predicting Bud Break Phenology

Buds of white spruce seed sources K20 and K24 from the temperate forest were more sensitive to photoperiod for phenological stages two (bud elongation, see **Table 3**) to five (bud scales ripped and needles exposed to air temperature), were more sensitive to spring frost occurrence for the phenological stages two (bud elongation) and three (bud swelling), and were more sensitive to growing degree-days for phenological stages four (translucent bud scales), five (bud scales ripped and needles exposed to air temperature), and six (complete bud flush) compared to the seed source J70 originating from the boreal mixedwood forest (**Table 5** and **Tables S20** to **S24**). Therefore, white spruce seed sources from the temperate forest required shorter day lengths, fewer growing degree-days, and opened their buds under a higher probability of spring frost occurrence than the seed source from the boreal mixedwood forest (**Table 5**).

**Table 5:** Results of generalized mixed binomial regressions for white spruce intraspecies variation in the timing of the bud break process.

Bud break	Frost probability (0–1)						Growing degree-days (°C, base temperature = 0°C)						Photoperiod (day length in hours)					
	Temperate forest			Boreal mixedwood forest			Temperate forest			Boreal mixedwood forest			Temperate forest			Boreal mixedwood forest		
	J70	K20	K24	J70	K20	K24	J70	K20	K24	J70	K20	K24	J70	K20	K24	J70	K20	K24
0 –> 2	**0.37 (141)**	**0.40 (140)**	**0.41 (139)**	**0.47 (141)**	**0.47 (141)**	**0.48 (141)**							**15.36 (141)**	**15.34 (140)**	**15.33 (140)**	**15.57 (143)**	**15.43 (139)**	**15.43 (139)**
2 –> 3	0.29 (143)	**0.44 (138)**	**0.37 (140)**	**0.25 (149)**	**0.39 (144)**	**0.33 (147)**	366 (146)	247 (135)	305 (142)	417 (151)	364 (147)	351 (146)	**15.54 (146)**	**15.21 (137)**	**15.38 (141)**	**15.81 (150)**	**15.70 (146)**	**15.66 (145)**
3 –> 4							**426 (150)**	**366 (146)**	**344 (144)**	424 (151)	369 (148)	351 (146)	15.67 (150)	**15.55 (146)**	**15.48 (144)**	**15.85 (151)**	**15.70 (146)**	**15.67 (146)**
4 –> 5							**430 (150)**	**406 (148)**	**411 (148)**	**492 (156)**	**470 (155)**	**452 (153)**	15.72 (152)	**15.64 (149)**	**15.66 (149)**	**15.97 (156)**	**15.95 (154)**	**15.89 (153)**
5 –> 6							**548 (158)**	**470 (153)**	**488 (154)**	**554 (162)**	**497 (157)**	**505 (157)**						
								Not better than the null model										
								Better than the null model but not the best model from the AICc model selection										
								Best model from AICc model selection										

The predicted value per environmental variable is shown at which the probability that a bud transitions to the next stage is 0.51 per seed source, while numbers in parentheses show day-of-year at which the transition is predicted to occur. Boldface type indicates coefficients that differ from zero, while colors indicate model performance evaluated based upon corrected Akaike's information criteria (AICc).

Since coefficients for the photoperiod models were stronger than those of the frost probability models, seed sources from the temperate forest were more sensitive to photoperiod than the local seed source originating from the boreal mixedwood forest (**Tables S20** and **S21**). This result is further supported since the frost probability model is incapable of predicting the observed earlier bud opening of seed sources from the temperate forest compared to the seed source from the boreal mixedwood forest whereas the photoperiod model more accurately predicts this difference in bud break timing (**Table 5**).

For the Norway spruce, the early emerging seed source K55 was more sensitive to the probability of spring frost occurrence for the first two transitions (**Table 6** and **Tables S25** to **S29**), was more sensitive to photoperiod for phenological stages one to five (**Table 6** and **Tables S25** to **S29**), and was more sensitive to growing degree-days for phenological stages four to six compared to later emerging seed sources K35, K39, and K50 (**Table 6** and **Tables S25** to **S29**). Therefore, the seed source K55 required shorter day lengths, fewer growing degree-days, and opened its buds under a higher probability of spring frost occurrence than late-emerging seed sources (**Table 6** and **Tables S25** to **S29**).

**Table 6:** Results of generalized mixed binomial regressions showing Norway spruce intraspecies variation in the timing of the bud break process.

	Frost probability (0–1)								Growing degree-days (°C, base temperature = 0°C)								Photoperiod (day length in hours)							
	Temperate forest				Boreal mixedwood forest				Temperate forest				Boreal mixedwood forest				Temperate forest				Boreal mixedwood forest			
	K35	K39	K50	K55	K35	K39	K50	K55	K35	K39	K50	K55	K35	K39	K50	K55	K35	K39	K50	K55	K35	K39	K50	K55
0 -> 2	0.27 (145)	0.28 (144)	0.24 (146)	0.32 (142)	**0.33 (146)**	**0.33 (146)**	**0.28 (148)**	**0.37 (145)**									**15.56 (145)**	15.54 (145)	15.61 (147)	**15.47 (144)**	**15.82 (149)**	**15.70 (146)**	**15.79(149)**	**15.64 (145)**
2 -> 3	**0.11 (154)**	0.09 (156)	0.10 (155)	0.16 (150)	**0.15 (155)**	**0.12 (157)**	**0.12 (157)**	**0.33 (146)**	503 (155)	534 (157)	516 (156)	448 (152)	506 (157)	528 (159)	516 (158)	468 (154)	**15.82(154)**	**15.86 (156)**	**15.86 (156)**	**15.71 (150)**	**15.95 (155)**	**15.97 (155)**	**15.96 (155)**	**15.90 (153)**
3 -> 4									**567 (159)**	**556 (158)**	**594 (161)**	**426 (150)**	**541 (161)**	**541 (161)**	**543 (161)**	**461 (154)**	**15.89 (159)**	**15.90 (159)**	**15.90 (159)**	**15.71 (150)**	**16.04 (159)**	**16.01 (156)**	**16.15 (166)**	**15.92 (154)**
4 -> 5									**569 (159)**	**591 (161)**	**516 (156)**	**505 (155)**	607 (166)	614 (166)	547 (161)	538 (160)	**15.91 (159)**	**15.94 (161)**	**15.84 (156)**	**15.79 (154)**	**16.09 (161)**	**16.07 (160)**	**16.05 (159)**	**16.12 (164)**
5 -> 6									613 (163)	644 (165)	587 (161)	472 (153)	636 (168)	662 (169)	607 (163)	470 (155)								
											Not better than the null model														
											Better than the null model but not the best model from AICc model selection														
											Best model from AICc model selection														

The predicted value per environmental variable is shown at which the probability that a bud transitions to the next stage is 0.51 per seed source, while numbers in parentheses show day-of-year at which the transition is predicted to occur. Boldface type indicates coefficients that differ from zero, while colors indicate model performance evaluated based upon corrected Akaike's information criteria (AICc).

### Heating Requirements for Bud Flush Based Upon a Degree-Day Threshold

The stage where needles are exposed to air temperate (stage five, see **Table 3**) is the stage that is conventionally analyzed (Hunter and Lechowicz, 1992; Hannerz, 1999; Snyder et al., 1999). Therefore, we used our mixed binomial regression model to predict the number of growing degree-days that were required for buds to reach that stage of bud phenology (stage five, see **Table 3**). At both plantation sites, white spruce's buds required fewer heating units to break compared to buds of black spruce and Norway spruce (**Table 4**). At the temperate forest site, buds of all three spruce species required fewer heating units to break compared to those at the boreal mixedwood forest site (**Table 4**).

At the intraspecies level, all three white spruce seed sources required on average 56 more heating units at the boreal mixedwood plantation to break compared to the temperate forest site. In both plantation sites, buds of the nonlocal southern white spruce seed sources K20 and K24 required on average 26 fewer heating units to break compared to buds of the northern seed source J70 (**Table 5**). All Norway spruce seed sources required more heating units in the boreal mixedwood forest than in the temperate forest to break their buds. At both sites, the Norway spruce seed source K55 required fewer heating units to break its buds compared to all remaining Norway spruce seed sources (**Table 6**).

## Discussion

### Inter- and Intraspecies Variation in the Timing of Bud Break

Bud flush started first for white spruces, followed by black spruces, and lastly, by Norway spruces, while the termination of bud flush ended first in white spruces, then in Norway spruces, and finally in black spruces. O'Reilly and Parker (1982), and Numainville and Desponts (2004) reported the same species ordering for the bud flush sequences. For each species, bud flush ended earlier in the temperate forest than in the boreal mixedwood forest plantation. The time required for buds to complete their leaf-out was within the 15–23 day range recorded for white spruces (16–19 days vs. 18 days reported by Rossi and Isabel, 2017), for black spruces (19–23 days vs. 15–23 days reported by Rossi and Bousquet, 2014; Sylvestro et al., 2019), and for Norway spruces (15–17 days vs. 18–23 days reported by Fløistad and Granhus, 2010). The shorter duration of black spruce bud flush in the boreal mixedwood relative to the temperate forest may result from an adaptation selected to maximize carbon gains during the shorter growing season of the north (Clark et al., 2014b; Sylvestro et al., 2019). Yet, the duration of bud flush of white spruce and Norway spruce was not faster in the boreal mixedwood forest compared to the temperate forest. Given that our study sites covered a small portion (47°–48° N) of continent-wide species ranges likely contributed to the observed decrease in clinal trend in bud flush with latitude. Still, earlier bud flush of nonlocal white spruce seed sources, when compared to the local white spruce seed source in the boreal mixedwood plantation, increased their probability of exposure to spring frosts, a result that was also reported in a transplant study conducted on 23 white spruce seed sources in Ontario (Canada) by Lu and Man (2011).

At both sites, three Norway spruce seed sources followed a similar bud break sequence (K35, K39 and K50) whereas the K55 seed source from Latvia completed its bud break ~10 days earlier. Since the Norway spruce is widely distributed in Europe, different tree populations are probably adapted to their local conditions (Matras, 2009; Chmura et al., 2018), thus, the earlier bud break from the Latvia seed source probably reflects a local adaptation. The similar bud break sequence from three different Norway spruce seed sources could suggest weak local adaptations regarding the bud break sequence or that the eastern Canadian boreal forest is so stressful to these three Norway spruce seed sources that it masked their difference in bud break sequence. Effectively, Klisz et al. (2019) showed that when climate is the most important factor determining the development of trees such as at the margin of a species range, the importance of local adaptation is reduced.

### Interspecies Variation in the Effect of Air Temperature and Photoperiod for Predicting Bud Break Phenology

White spruces exhibited a clear change in sensitivity to air temperature variables, consistent with our hypothesis. Its first stages were driven by spring frost occurrence, whereas its intermediate and final stages were driven by growing degree-days. Therefore, the white spruce shifts its strategy from frost avoidance in the early stages to maximize the growing season length by flushing buds more rapidly with increasing air temperature for the intermediate and final stages. Specifically, spring frost occurrence was most important in controlling bud flush in the boreal forest, where this phenomenon was the most frequent. In contrast, sensitivity to photoperiod in the temperate forest could replace the lower spring frosts occurrence to trigger bud flush. In environments where spring frosts rarely damage trees severely, day length would be the environmental variable driving bud break timing. Both the black spruce and the Norway spruce, however, were less sensitive to spring frosts occurrence, but they were sensitive to growing degree-days for their intermediate and final stages of bud phenology. The black spruce was the least sensitive species to both growing degree-days and spring frost occurrence, but its late bud flush prevented damage to buds from spring frost events and its faster completion of the bud break process could maximize growing season length. Thus, late-emerging species may wait for a low frost frequency before opening their buds, but hasten their development, which maximizes the growing season length. In contrast, early-emerging species may develop their buds earlier, but more slowly to keep the leaf primordia protected by the bud scales while frost probability is high.

Since our study is only empirical, future experiments also aiming to disentangle the importance of frost, photoperiod and growing degree-days on the bud break sequence of spruces should be conducted and compared to our results. Still, frost probability in late spring represents a just simplification of the evolutionary trade-off imposed on boreal and temperate trees by the freezing temperatures. Even if laboratory studies suggest that frost events of low intensity have minimal impact on the bud break sequence of spruces since the dehardening threshold values preventing frost damage to buds decreased from −10°C at the swelling stage to −5°C at the bud break stage (Glerum, 1973; Repo, 1992; Bigras and Hébert, 1996), it contradicts with the frost damage to buds or newly formed foliage we observed (gray-black buds or brown-white needles leaning downward, **Figure S3**) after a night frost of −2°C (data not shown), which was also reported by Cannell (1984). Frost damage to buds probably follows a dynamic process where frost damage occurs when the heat loss by trees at night exceeds the heat accumulated during the day. Since laboratory studies likely grew seedlings to air temperatures above those observed in the field, field trees probably accumulated less heat during the day and could therefore be damaged by less intense frost events. Since the synchrony between spring frosts and the bud break was shown to be an important aspect of tree fitness (Vitra et al., 2017), the probability of spring frost could better represent the evolutionary pressure imposed by late-spring frosts on the survival of trees compared to growing degree-days.

### Intraspecies Variation in the Effect of Air Temperature and Photoperiod for Predicting Bud Break Phenology

We tried to quantify the capacity of nonlocal white and Norway spruce seed sources to adapt when transplanted to a new climate that was used as a proxy for climate change. We showed that nonlocal white spruce seed sources originating from the southern temperate forest opened their buds earlier than did the local seed source from the boreal mixedwood forest. Therefore, nonlocal southern seed sources could only partly adapt to their new climate, which is consistent with our hypothesis. Specifically, early stages of bud phenology of the nonlocal (southern) white spruce seed sources were more sensitive to photoperiod compared to the local seed source and were also more sensitive to growing degree-days for their intermediate stages of bud phenology. Our results for the black spruce seed source originating from the boreal forest, where spring frost probability is high, showed that bud flush was slightly more sensitive to air temperature than to photoperiod. We propose a general explanation driving the leaf-out process in spring. On one hand, tree populations that are growing in environments where spring frost is frequent and likely throughout the growing season, as is the case in the boreal forest, have adapted to the risk of frost damage. Accordingly, onset of bud flush is driven by frost probability. On the other hand, tree populations that are growing in environments where air temperature warms more rapidly, and where spring frost probability decreases quickly, have adapted to a photoperiod-triggered leaf-out, concomitant with the decrease in spring frost probability.

As in previous studies, we found a large within-tree variation in bud break timing (Rousi and Heinonen, 2007; Sylvestro et al., 2019) that probably results from an adaptation to limit the damage caused by spring frosts. The late bursting of some buds ensures that trees can still grow even if some buds were damaged by spring frosts. However, this large variation in bud break timing caused some problems when we predicted the average date buds transit from a phenological stage to the next because the predicted date at which buds reached late transitions could have been predicted prior to the dates at which buds reached earlier transitions (**Figure 4**). By analyzing each step separately instead of analyzing each step in a single model, we might have increased the overlap between stages (Clark et al., 2014a; Clark et al., 2014b). The overlap problem was mostly observed for Norway spruce seed sources (K50 and K55). Since the predictions were based upon the probability (0.51) that buds transited toward the next stage, the probability that buds did not transit was still of 0.49, which might have increased the overlap problem between successive stages. Had we chosen a different threshold value; the proportion of overlap would have also changed. We confirm that at the bud level no late stages were registered before earlier ones therefore, the overlap problem is likely due to the large variation in bud break timing between trees and to the smaller sample size at the seed source level. Still, errors were of fewer days than our sampling interval. Given the logistical constraints, it was not possible to sample at a shorter time interval. Since it is common for bud phenology data to show some overlap between successive stages (Clark et al., 2014a), these rare problems unlikely discredit the general conclusion of our study but call for increasing studies analyzing each stage of the bud break process, which for now are still scarce. We acknowledge that Clark et al. (2014a; 2014b) developed a continuous development model (CDM) consisting of a Multinomial Hidden Markov Chain within a Bayesian framework to analyze the bud break process, but the greater complexity of running this model compared to running separated binomial regressions informed our choice.

### Heating Requirements for Bud Flush Based on Growing Degree-Day Thresholds

The bursting of buds is a phenotypic trait that is under the control of both genetic and phenotypic plasticity (Worrall and Mergen, 2006; Yakovlev et al., 2006; Pelgas et al., 2011; Rossi and Bousquet, 2014). However, the share of both components (genetics and phenotypic plasticity) is likely species- and population-dependent, thus, the capacity of different tree populations to vary their timing of bud break according to changes in climate will vary across tree populations and species. With heating requirement for bud flush varying between 77 and 1278 growing degree-days, the Norway spruce is a spruce species showing high variations in timing of bud break compared to the white spruce, which required between 233 and 360 degree-days (Man and Lu, 2010; Man et al., 2016; Man et al., 2017) and the black spruce, which required between 284 and 514 growing degree-days (Bronson et al., 2009; Antonucci et al., 2015; Man et al., 2016). In our study, the number of GDDs that were required for white (450 GDD) and black spruce, (626 GDD) bud flush was slightly above their previously reported upper range whereas the Norway spruce (572 GDD) was in the middle of its reported range.

Blum (1988) developed the hypothesis that populations of trees growing in colder sites (north) would require fewer heating units to flush their buds compared to southern populations. Similar results were reported by Sylvestro et al. (2019). In the present study, the heating requirement for all three spruce species were higher at the boreal mixedwood plantation site and contrast with Blum's (1988) hypothesis. However, our results are consistent with our hypothesis that frost probability outperformed degree-day sums to trigger onset of bud flush. We suggest that in sites where spring frost probability is high, onset of bud flush is postponed, which also increases their heating requirement whereas in sites where spring frost frequency is low, buds flush sooner, which decreases their heating requirement. Yet, complexity arises because air temperature increases more slowly in the boreal site. Even if bud flush is later in terms of calendar days, accumulated degree-days can still be lower compared to temperate sites where air temperature increases more rapidly. We suggest that the intraspecies variation in heating requirement found in previous studies probably represents the variation in spring frost probability that is experienced by the different tree populations. Even if traditional studies have used growing degree-days to predict bud flush, we have advanced the idea that spring frost occurrence is the main driver of spruce bud flush. In fact, Kollas et al. (2014) showed that the environmental driver common to both altitudinal and latitudinal species range limitation was frost risk during leaf-out. Frost probability was also shown to drive the leaf-out process of various tree species along an altitudinal gradient in Switzerland (Lenz et al., 2016) and on the Tibetan Plateau (Wang et al., 2019). Therefore, ecophysiological models of bud break should include spring frost variables.

### Adaptation Capacity to Spring Frosts in the Context of Climate Change

Spring frosts are daily events that require a set of specific meteorological conditions, such as a night without wind and clouds, to occur (Laughlin and Kalma, 1987; Lindkvist and Lindqvist, 1997; Chung et al., 2006). These specific conditions are hardly modeled with accuracy by climate change models. Since climate change already increased the global mean annual temperature by 1°C (Allen et al., 2018) but that spring frosts still occur frequently in boreal mixedwood forests (see Study site section), climate warming over the next 50 years will unlikely decrease the future frequency and intensity of spring frosts enough to limit their impact on tree growth. Consistent with previous studies assessing the impact of climate change on bud phenology (Polgar et al., 2013; Olsson et al., 2017), our results suggest that bud flush of spruce species would hasten in the future because higher air temperatures will accelerate transitions of the temperature-sensitive stages of bud phenology. Yet, photoperiod-sensitive stages of bud phenology should limit premature leaf-out and become a more prominent safety mechanism preventing frost damage. Experimental temperature manipulations have shown that the bud break sequence of boreal tree species was completed earlier when they were artificially heated, but that faster bud break was restricted to the last stages of bud phenology, which are the stages we demonstrated to be less sensitive to photoperiod (Rossi, 2015; Rossi and Isabel, 2016; Rossi and Isabel, 2017). It was also shown that the first stages of bud phenology were under stronger genetic control compared to the last phenological stages (Perrin et al., 2017). Under a warmer climate, we would expect earlier bud flush in spruce species that are growing in boreal mixedwood forests, since they are more sensitive to air temperature compared to spruce species growing in southern temperate forests. We also expected that the black spruce, which is less photoperiod-sensitive than the white spruce, would advance its bud phenology more rapidly, which would increase its risk of frost damage. Overall, convergence of bud phenology across spruce species is to be expected, an observation that has also been reported over altitudinal gradients (Chen et al., 2018; Vitasse et al., 2018).

Our results can also guide climate-smart forestry practices aiming to manage the boreal-temperate forest ecotone in a sustainable way (Nagel et al., 2017; Verkerk et al., 2020). The long (60–100 years) rotation length of spruce plantations established in the boreal mixedwood forest paired with the fast rate of climate warming ensures that trees currently planted will be growing in a warmer climate than the current climate. However, planting for the future first requires the survival of the planted seedlings in their early years. Accordingly, photoperiod-sensitive seed sources such as the southern with spruce seed sources consist in a poor choice for establishing productive plantations in the boreal mixedwood forest since these seed sources will likely be frequently damaged by spring frosts. Local seed sources better adapted to prevent damage from spring frost consist in a better choice for establishing productive plantations. Since the black spruce is the spruce species that burst its buds the latest, it is the least likely species to be damaged by spring frosts, therefore, it currently represents the best species to plant in spring frost-prone environments. However, the warming of air temperature paired with the black spruce low photoperiod sensitivity should hasten its future bud burst and increase its exposure to spring frost, thus, probably reducing its future productivity. Interestingly, we showed that assisted migration and the use of foreign species might not always be a good option for increasing tree productivity, mostly when extreme events such as spring frosts can importantly damage trees.

## Conclusion

Our novel approach allowed us to identify the probability of spring frost occurrence as the driver of bud break onset, while growing degree-days drove the intermediate and final phenological stages of bud break. Incorporating this change in sensitivity to climate along the bud break sequence should increase the realism of process-based models of bud burst in temperate and boreal tree species. Further, its inclusion should increase the accuracy of predicting the response of bud break phenology to climate change and help to plan forest management practices that could mitigate the negative effects of climate change on forest productivity.

## Data Availability Statement

The raw data supporting the conclusions of this article are stored as a research dataset on the Mendeley reference management software http://dx.doi.org/10.17632/2t4b33ftst.1.

## Author Contributions

The experimental plantations were designed and established in 2002 by FT. BM developed the hypotheses to be tested, determined the sampling procedures, did the field work, analyzed the data, and wrote the first draft of the manuscript. YB and MS helped to develop the hypotheses, the methods, the statistical analyses, and all authors contributed substantially to writing the final manuscript.

## Funding

This research project was funded through a grant provided by the Natural Science and Engineering Research Council of Canada (NSERC) to FT and colleagues.

## Conflict of Interest

The authors declare that the research was conducted in the absence of any commercial or financial relationships that could be construed as a potential conflict of interest.
