# Supplementary material for: Probability of Spring Frosts, Not Growing Degree-Days, Drives Onset of Spruce Bud Burst in Plantations at the Boreal-Temperate Forest Ecotone
Source: Front Plant Sci. 2020 Jul 22;11:1031. doi: 10.3389/fpls.2020.01031 (PMC7396537; doi:10.3389/fpls.2020.01031)
Supplement: Supplementary file 1 [file DataSheet_1.docx]

Supplementary Material


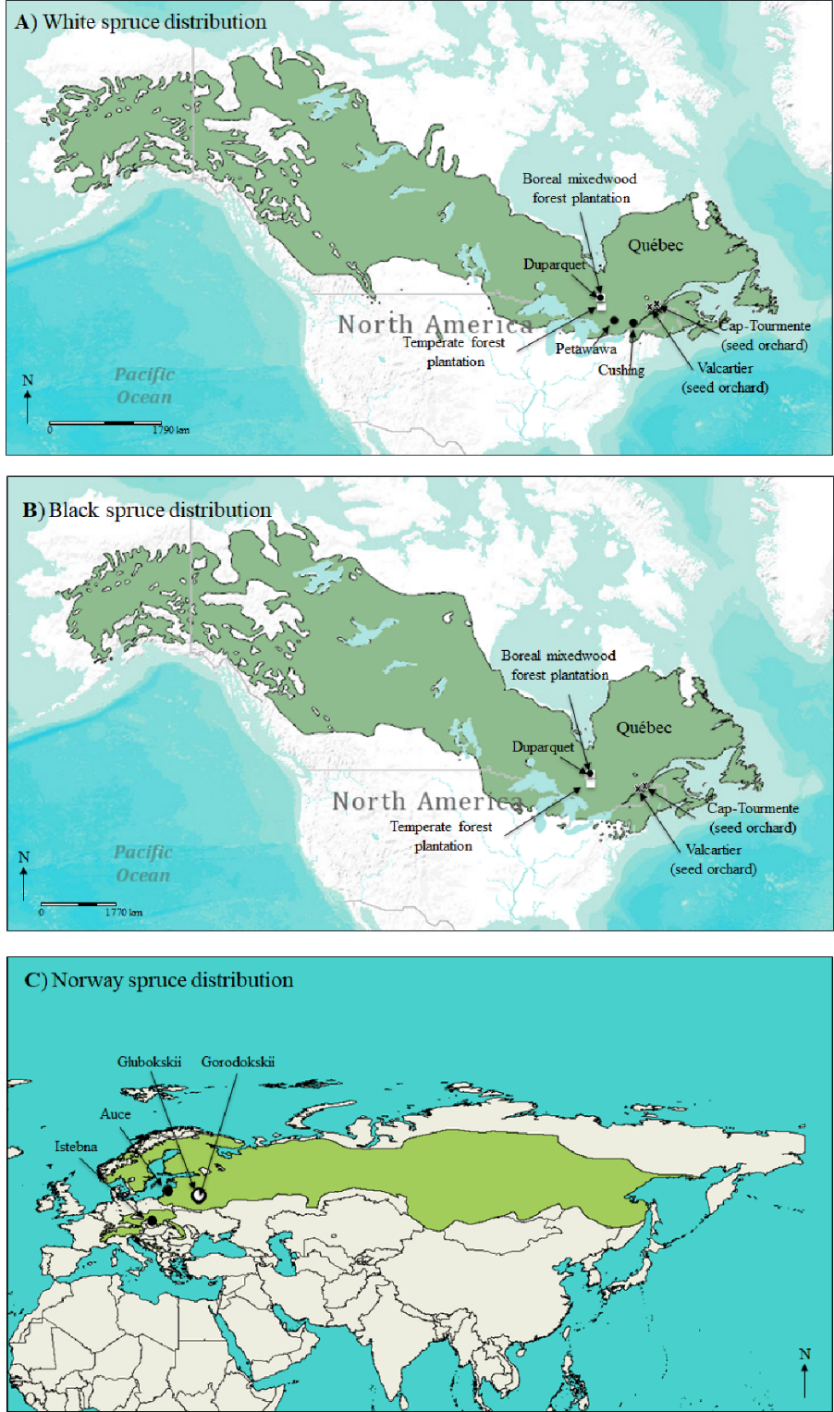


Figure S1 Maps showing the locations of the seed sources within each species range. Species distribution data were retrieved from data basin <https://databasin.org/datasets/0dd88c1ae391403698fbc9d457154bdc> for white spruce and black spruce and from the European Forest Genetic Resources Programme (EUFORGEN) <http://www.euforgen.org/species/picea-abies/> for Norway spruce.


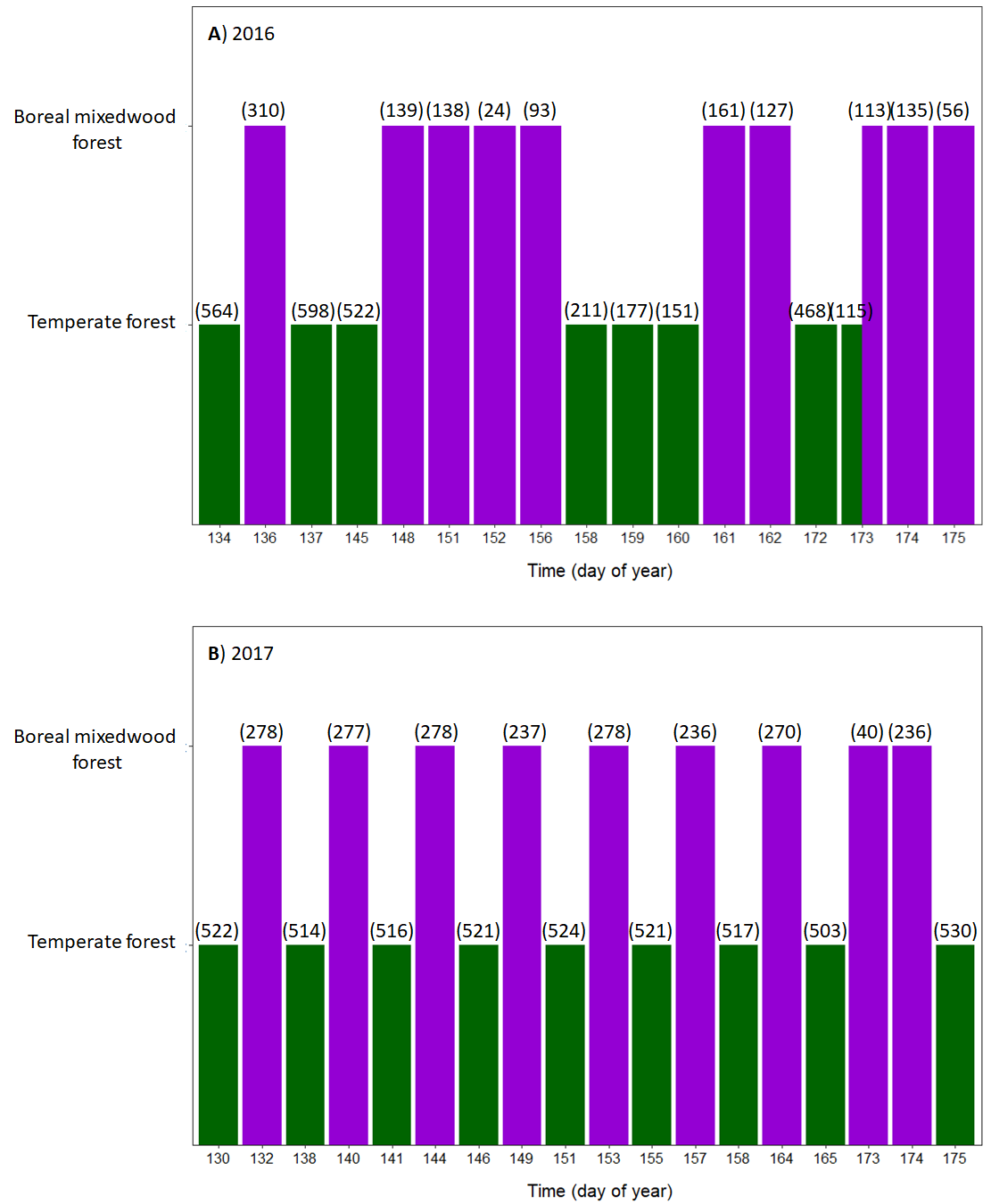
Figure S2 Timeline showing the dates (in day of year) and the number of bud observations (in parentheses) at which buds were observed at each plantation sites (green represent temperate forest plantation and violet represents the boreal


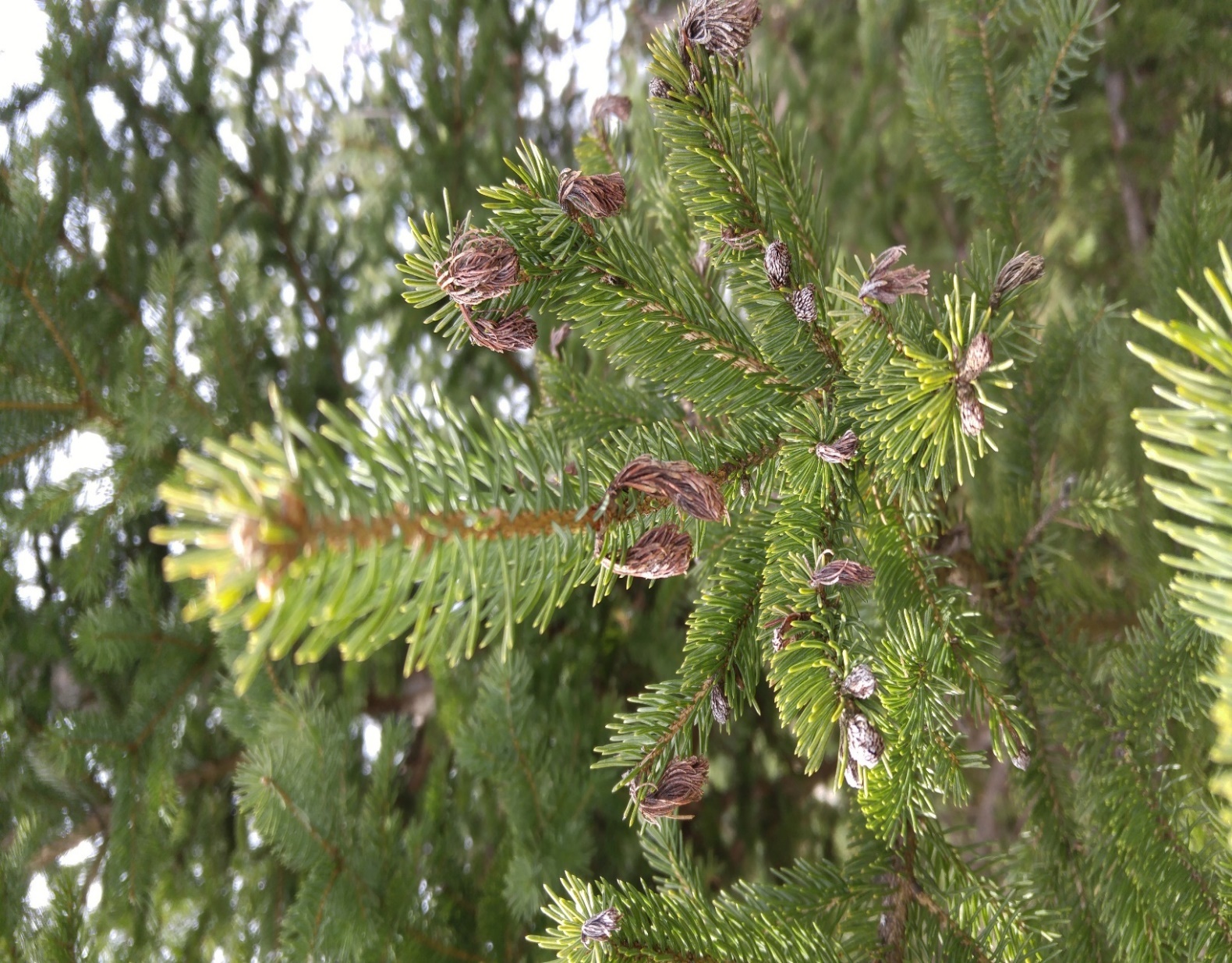


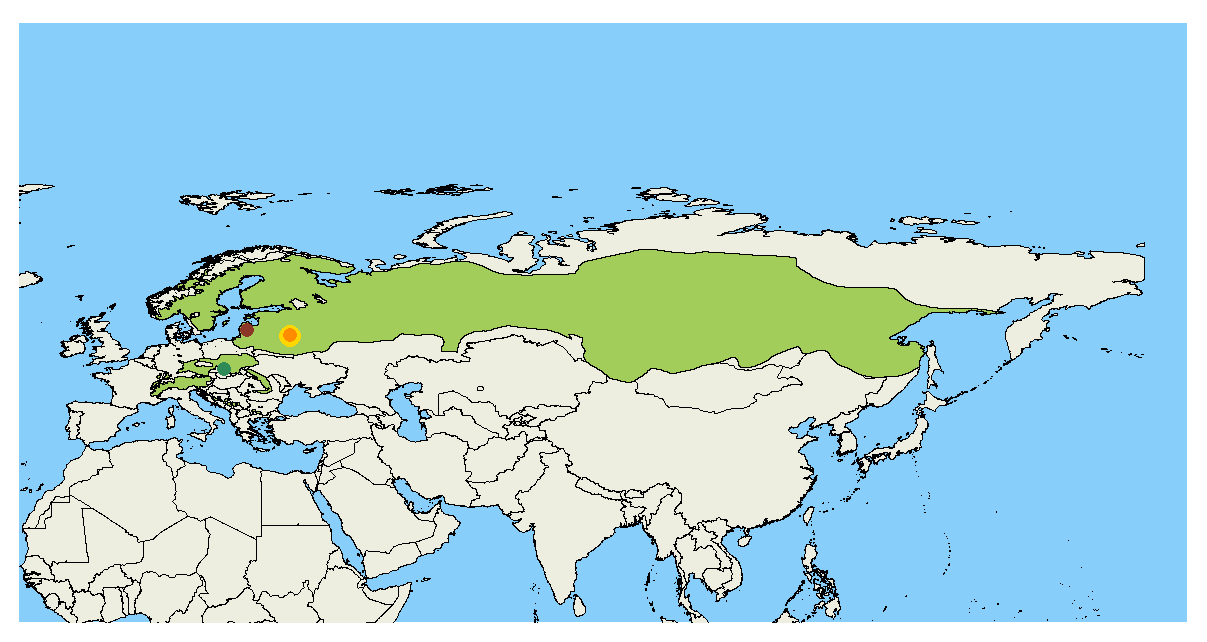
Figure S3 Spring frost damage on newly formed needles (brown-grey needles leaning downward).

**C**) Norway spruce distribution

Auce

Istebna

Glubokskii

Gorodokskii

| **Site** | **iButtons** | **# Days (period)** | **Years** | **Climate Variables** | **Adjusted *R^2^*** | **# Missing dates (period)** |
| --- | --- | --- | --- | --- | --- | --- |
| Temperate | 1 | 99 (137-235) | 2016 | Minimum temp | 0.81 | 7 (130-136) |
| Temperate | 1 | 95 (146-240) | 2017 | Minimum temp | 0.81 | 16 (130-145) |
| Temperate | 1 | 99 (137-235) | 2016 | Mean temp | 0.91 | 7 (130-136) |
| Temperate | 1 | 95 (146-240) | 2017 | Mean temp | 0.91 | 16 (130-145) |
| Temperate | 1 | 99 (137-235) | 2016 | Maximum temp | 0.85 | 7 (130-136) |
| Temperate | 1 | 95 (146-240) | 2017 | Maximum temp | 0.85 | 16 (130-145) |
| Temperate | 2 | 99 (137-235) | 2016 | Minimum temp | 0.81 | 7 (130-136) |
| Temperate | 2 | 95 (146-240) | 2017 | Minimum temp | 0.81 | 16 (130-145) |
| Temperate | 2 | 99 (137-235) | 2016 | Mean temp | 0.91 | 7 (130-136) |
| Temperate | 2 | 95 (146-240) | 2017 | Mean temp | 0.91 | 16 (130-145) |
| Temperate | 2 | 99 (137-235) | 2016 | Maximum temp | 0.89 | 7 (130-136) |
| Temperate | 2 | 95 (146-240) | 2017 | Maximum temp | 0.89 | 16 (130-145) |
| Temperate | 3 | 96 (145-240) | 2016 | Minimum temp | 0.83 | 15 (130-144) |
| Temperate | 3 | 95 (146-240) | 2017 | Minimum temp | 0.83 | 16 (130-145) |
| Temperate | 3 | 96 (145-240) | 2016 | Mean temp | 0.91 | 15 (130-144) |
| Temperate | 3 | 95 (146-240) | 2017 | Mean temp | 0.91 | 16 (130-145) |
| Temperate | 3 | 96 (145-240) | 2016 | Maximum temp | 0.82 | 15 (130-144) |
| Temperate | 3 | 95 (146-240) | 2017 | Maximum temp | 0.82 | 16 (130-145) |

Table S1 Results of the linear regression between daily minimum, mean and maximum air temperature measured with the temperature loggers (iButtons) and the temperature simulated with the software Biosim 10 at the plantation in temperate forest. The P-value for each regression is below the significance level of 0.05.

Table S2 Results of the linear regression between daily minimum, mean and maximum air temperature measured with the temperature loggers (iButtons) and the temperature simulated with the software Biosim 10 at the plantation in boreal mixedwood forest. The P-value for each regression is below the significance level of 0.05.

| **Site** | **iButtons** | **# of days (period)** | **Years** | **Climate variables** | **Adjusted *R^2^*** | **Missing dates (period)** |
| --- | --- | --- | --- | --- | --- | --- |
| Boreal | 1 | 80 (143-222) | 2016 | Minimum temp | 0.86 | 13 (130-142) |
| Boreal | 1 | 97 (143-239) | 2017 | Minimum temp | 0.86 | 13 (130-142) |
| Boreal | 1 | 80 (143-222) | 2016 | Mean temp | 0.93 | 13 (130-142) |
| Boreal | 1 | 97 (143-239) | 2017 | Mean temp | 0.93 | 13 (130-142) |
| Boreal | 1 | 80 (143-222) | 2016 | Maximum temp | 0.92 | 13 (130-142) |
| Boreal | 1 | 97 (143-239) | 2017 | Maximum temp | 0.92 | 13 (130-142) |
| Boreal | 2 | 82 (141-222) | 2016 | Minimum temp | 0.77 | 11 (130-140) |
| Boreal | 2 | 97 (143-239) | 2017 | Minimum temp | 0.77 | 13 (130-142) |
| Boreal | 2 | 82 (141-222) | 2016 | Mean temp | 0.92 | 11 (130-140) |
| Boreal | 2 | 97 (143-239) | 2017 | Mean temp | 0.92 | 13 (130-142) |
| Boreal | 2 | 82 (141-222) | 2016 | Maximum temp | 0.74 | 11 (130-140) |
| Boreal | 2 | 97 (143-239) | 2017 | Maximum temp | 0.74 | 13 (130-142) |
| Boreal | 3 | 89 (140-228) | 2016 | Minimum temp | 0.85 | 10 (130-139) |
| Boreal | 3 | 97 (143-239) | 2017 | Minimum temp | 0.85 | 13 (130-142) |
| Boreal | 3 | 89 (140-228) | 2016 | Mean temp | 0.94 | 10 (130-139) |
| Boreal | 3 | 97 (143-239) | 2017 | Mean temp | 0.94 | 13 (130-142) |
| Boreal | 3 | 89 (140-228) | 2016 | Maximum temp | 0.86 | 10 (130-139) |
| Boreal | 3 | 97 (143-239) | 2017 | Maximum temp | 0.86 | 13 (130-142) |

Table S3 List of the 31 candidate models tested to determine the best air temperature predictors of the transition between phenological stages.

| **Model ID** | **Predictor variables** | **Interactions** |
| --- | --- | --- |
| 1 | DOY (Null) | NA |
| 2 | Daily max temp. | NA |
| 3 | Daily mean temp. | NA |
| 4 | Daily min temp. | NA |
| 5 | GDD (max) | NA |
| 6 | GDD (mean) | NA |
| 7 | Frost prob. | NA |
| 8 | Daily max temp.; Species | NA |
| 9 | Daily mean temp.; Species | NA |
| 10 | Daily min temp.; Species | NA |
| 11 | GDD (max); Species | NA |
| 12 | GDD (mean); Species | NA |
| 13 | Frost prob; Species | NA |
| 14 | Daily max temp.; Site | NA |
| 15 | Daily mean temp.; Site | NA |
| 16 | Daily min temp.; Site | NA |
| 17 | GDD (max); Site | NA |
| 18 | GDD (mean); Site | NA |
| 19 | Frost prob; Site | NA |
| 20 | Daily max temp.; Species; Site | NA |
| 21 | Daily mean temp.; Species; Site | NA |
| 22 | Daily min temp.; Species; Site | NA |
| 23 | GDD (max); Species; Site | NA |
| 24 | GDD (mean); Species; Site | NA |

(Table S3 suite)

| **Model ID** | **Predictor variables** | **Interactions** |
| --- | --- | --- |
| 25 | Frost prob; Species; Site | NA |
| 26 | Daily max temp.; Species; Site | Daily max temp. * Species;  Daily max temp. * Site |
| 27 | Daily mean temp.; Species; Site | Daily mean temp * Species;  Daily mean temp * Site |
| 28 | Daily min temp.; Species; Site | Daily min temp. * Species;  Daily min temp. * Site |
| 29 | GDD (max); Species; Site | GDD (max) * Species;  GDD (max) * Site |
| 30 | GDD (mean); Species; Site | GDD (mean) * Species;  GDD (mean) * Site |
| 31 | Frost prob; Species; Site | Frost prob * Species;  Frost prob * Site |

Table S4 List of the six candidate models that were tested to determine the best photoperiod predictors of transitions between phenological stages.

| **Model ID** | **Predictor variables** | **Interactions** |
| --- | --- | --- |
| 1 | DOY (Null) | NA |
| 2 | Photoperiod | NA |
| 3 | Photoperiod; Species | NA |
| 4 | Photoperiod; Site | NA |
| 5 | Photoperiod; Species; Site | NA |
| 6 | Photoperiod; Species; Site | Photoperiod * Species;  Photoperiod * Site |

Table S5 Results of the AICc model selection to determine the best combination of air temperature variables for predicting the transition from stage zero to stage two of spruce bud phenology, bold indicated the selected models and numbers in parentheses before each model refer to the candidate models before selection (Table S3).

| **Models** | **AICc** | **Delta AICc** | **AICc weight** | | **AICc cum. weight** |
| --- | --- | --- | --- | --- | --- |
| **(31) Frost prob.; Species; Site, Interactions** | **3342.35** | **0.00** | | **1.00** | **1.00** |
| (25) Frost prob.; Species; Site | 3510.61 | 168.26 | | 0.00 | 1.00 |
| (13) Frost prob.; Species | 3525.47 | 183.11 | | 0.00 | 1.00 |
| (19) Frost prob.; Site | 3582.67 | 240.32 | | 0.00 | 1.00 |
| (7) Frost prob. | 3603.42 | 261.07 | | 0.00 | 1.00 |
| (1) Day of year | 3861.83 | 519.48 | | 0.00 | 1.00 |
| (29) GDD max; Species; Site, Interactions | 3862.54 | 520.19 | | 0.00 | 1.00 |
| (30) GDD mean; Species; Site, Interactions | 3933.03 | 590.68 | | 0.00 | 1.00 |
| (17) GDD max; Site | 3980.74 | 638.39 | | 0.00 | 1.00 |
| (11) GDD max; Species | 3995.71 | 653.35 | | 0.00 | 1.00 |
| (5) GDD max | 4020.06 | 677.71 | | 0.00 | 1.00 |
| (24) GDD mean; Species; Site | 4076.76 | 734.41 | | 0.00 | 1.00 |
| (12) GDD mean; Species | 4077.00 | 734.65 | | 0.00 | 1.00 |
| (6) GDD mean | 4117.77 | 775.42 | | 0.00 | 1.00 |
| (18) GDD mean; Site | 4119.66 | 777.31 | | 0.00 | 1.00 |
| (27) Mean daily T. °C; Species; Site; Interactions | 5706.09 | 2363.73 | | 0.00 | 1.00 |
| (21) Mean daily T. °C; Species | 5747.40 | 2405.04 | | 0.00 | 1.00 |
| (15) Mean daily T. °C; Site | 5751.30 | 2408.95 | | 0.00 | 1.00 |
| (9) Mean daily T. °C; Species | 5751.80 | 2409.45 | | 0.00 | 1.00 |
| (3) Mean daily T. °C | 5758.77 | 2416.42 | | 0.00 | 1.00 |

(Table S5 suite)

| **Models** | **AICc** | **Delta AICc** | **AICc weight** | **AICc cum. weight** |
| --- | --- | --- | --- | --- |
| (28) Min daily T. °C; Species; Site; Interactions | 5826.21 | 2483.86 | 0.00 | 1.00 |
| (26) Max daily T. °C; Species; Site; Interactions | 5940.38 | 2598.02 | 0.00 | 1.00 |
| (8) Max daily T. °C; Species | 5951.48 | 2609.13 | 0.00 | 1.00 |
| (2) Max daily T. °C | 5952.15 | 2609.80 | 0.00 | 1.00 |
| (14) Max daily T. °C; Site | 5953.01 | 2610.66 | 0.00 | 1.00 |
| (20) Max daily T. °C; Species; Site | 5953.02 | 2610.67 | 0.00 | 1.00 |
| (22) Min daily T. °C; Species; Site | 5997.91 | 2655.56 | 0.00 | 1.00 |
| (16) Min daily T. °C; Site | 6001.03 | 2658.68 | 0.00 | 1.00 |
| (10) Min daily T. °C; Species | 6021.29 | 2678.94 | 0.00 | 1.00 |
| (4) Min daily T. °C | 6029.29 | 2686.94 | 0.00 | 1.00 |
| (23) GDD max; Species; Site | NA | NA | NA | NA |

Table S6: Results of the AICc model selection to determine the best combination of air temperature variables for predicting the transition from stage two to stage three of spruce bud phenology, bold indicated the selected models and numbers in parentheses before each model refer to the candidate models before selection (Table S3).

| **Models** | **AICc** | **Delta AICc** | **AICc weight** | **AICc cum. weight** |
| --- | --- | --- | --- | --- |
| **(13) Frost prob.; Species** | **2244.30** | **0.00** | **0.57** | **0.57** |
| **(31) Frost prob.; Species; Site; Interactions** | **2246.16** | **1.86** | **0.22** | **0.79** |
| **(25) Frost prob.; Species; Site** | **2246.31** | **2.01** | **0.21** | **1.00** |
| (29) GDD max; Species; Site; Interactions | 2260.66 | 16.36 | 0.00 | 1.00 |
| (30) GDD mean; Species; Site; Interactions | 2268.96 | 24.67 | 0.00 | 1.00 |
| (24) GDD mean; Species; Site | 2282.31 | 38.01 | 0.00 | 1.00 |
| (12) GDD mean; Species | 2286.54 | 42.25 | 0.00 | 1.00 |
| (23) GDD max; Species; Site | 2305.66 | 61.37 | 0.00 | 1.00 |
| (11) GDD max; Species | 2348.35 | 104.05 | 0.00 | 1.00 |
| (1) Day of year | 2366.18 | 121.88 | 0.00 | 1.00 |
| (7) Frost prob. | 2390.25 | 145.95 | 0.00 | 1.00 |
| (19) Frost prob.; Site | 2392.06 | 147.76 | 0.00 | 1.00 |
| (6) GDD mean | 2420.85 | 176.56 | 0.00 | 1.00 |
| (18) GDD mean; Site | 2421.12 | 176.83 | 0.00 | 1.00 |
| (28) Min daily T °C; Species; Site; Interactions | 2807.79 | 563.49 | 0.00 | 1.00 |
| (27) Mean daily T °C; Species; Site; Interactions | 2876.97 | 632.68 | 0.00 | 1.00 |
| (22) Min daily T °C; Species; Site | 2887.51 | 643.21 | 0.00 | 1.00 |
| (10) Min daily T °C; Species | 2887.80 | 643.50 | 0.00 | 1.00 |

(Table S6 suite)

| **Models** | **AICc** | **Delta AICc** | **AICc weight** | **AICc cum. weight** |
| --- | --- | --- | --- | --- |
| (26) Max daily T °C; Species; Site; Interactions | 2897.44 | 653.14 | 0.00 | 1.00 |
| (21) Mean daily T °C; Species; Site | 2899.96 | 655.67 | 0.00 | 1.00 |
| (20) Max daily T °C; Species; Site | 2900.11 | 655.81 | 0.00 | 1.00 |
| (9) Mean daily T °C; Species | 2903.15 | 658.86 | 0.00 | 1.00 |
| (8) Max daily T °C; Species | 2903.79 | 659.50 | 0.00 | 1.00 |
| (4) Min daily T °C | 2948.31 | 704.01 | 0.00 | 1.00 |
| (16) Min daily T °C; Site | 2949.44 | 705.14 | 0.00 | 1.00 |
| (15) Mean daily T °C; Site | 2960.00 | 715.71 | 0.00 | 1.00 |
| (14) Max daily T °C; Site | 2960.21 | 715.91 | 0.00 | 1.00 |
| (3) Mean daily T °C | 2960.43 | 716.13 | 0.00 | 1.00 |
| (2) Max daily T °C | 2960.93 | 716.63 | 0.00 | 1.00 |
| (17) GDD max; Site | NA | NA | NA | NA |
| (5) GDD max | NA | NA | NA | NA |

Table S7 Results of the AICc model selection to determine the best combination of air temperature variables for predicting the transition from stage three to stage four of spruce bud phenology, bold indicated the selected models and numbers in parentheses before each model refer to the candidate models before selection (Table S3).

| **Models** | **AICc** | **Delta AICc** | **AICc weight** | **AICc cum. weight** |
| --- | --- | --- | --- | --- |
| **(12) GDD mean; Species** | **1766.43** | **0.00** | **0.56** | **0.56** |
| **(24) GDD mean; Species; Site** | **1767.87** | **1.45** | **0.27** | **0.83** |
| **(30) GDD mean; Species; Site; Interactions** | **1769.28** | **2.85** | **0.13** | **0.97** |
| (23) GDD max; Species; Site | 1772.67 | 6.24 | 0.02 | 0.99 |
| (29) GDD max; Species; Site; Interactions | 1775.56 | 9.14 | 0.01 | 1.00 |
| (11) GDD max; Species | 1803.13 | 36.70 | 0.00 | 1.00 |
| (31) Frost prob.; Species; Site; Interactions | 1853.69 | 87.26 | 0.00 | 1.00 |
| (6) GDD mean | 1873.76 | 107.34 | 0.00 | 1.00 |
| (18) GDD mean; Site | 1875.45 | 109.03 | 0.00 | 1.00 |
| (17) GDD max; Site | 1878.87 | 112.44 | 0.00 | 1.00 |
| (5) GDD max | 1886.48 | 120.05 | 0.00 | 1.00 |
| (25) Frost prob.; Species; Site | 1899.68 | 133.25 | 0.00 | 1.00 |
| (13) Frost prob.; Species | 1905.39 | 138.96 | 0.00 | 1.00 |
| (1) Day of year | 1905.80 | 139.38 | 0.00 | 1.00 |
| (19) Frost prob.; Site | 1974.29 | 207.87 | 0.00 | 1.00 |
| (7) Frost prob. | 1983.20 | 216.77 | 0.00 | 1.00 |
| (26) Max daily T °C; Species; Site; Interactions | 2120.31 | 353.88 | 0.00 | 1.00 |
| (27) Mean daily T °C; Species; Site; Interactions | 2122.52 | 356.10 | 0.00 | 1.00 |

(Table S7 suite)

| **Models** | **AICc** | **Delta AICc** | **AICc weight** | **AICc cum. weight** |
| --- | --- | --- | --- | --- |
| (28) Min daily T °C; Species; Site; Interactions | 2124.11 | 357.69 | 0.00 | 1.00 |
| (22) Min daily T °C; Species; Site | 2136.55 | 370.13 | 0.00 | 1.00 |
| (21) Mean daily T °C; Species; Site | 2142.08 | 375.65 | 0.00 | 1.00 |
| (10) Min daily T °C; Species | 2143.70 | 377.28 | 0.00 | 1.00 |
| (20) Max daily T °C; Species; Site | 2143.90 | 377.48 | 0.00 | 1.00 |
| (16) Min daily T °C; Site | 2145.15 | 378.72 | 0.00 | 1.00 |
| (9) Mean daily T °C; Species | 2147.04 | 380.61 | 0.00 | 1.00 |
| (8) Max daily T °C; Species | 2148.12 | 381.69 | 0.00 | 1.00 |
| (15) Mean daily T °C; Site | 2149.31 | 382.88 | 0.00 | 1.00 |
| (14) Max daily T °C; Site | 2151.04 | 384.61 | 0.00 | 1.00 |
| (4) Min daily T °C | 2153.78 | 387.36 | 0.00 | 1.00 |
| (3) Mean daily T °C | 2156.02 | 389.59 | 0.00 | 1.00 |
| (2) Max daily T °C | 2157.00 | 390.57 | 0.00 | 1.00 |

Table S8 Results of the AICc model selection to determine the best combination of air temperature variables for predicting the transition from stage four to stage five of spruce bud phenology, bold indicated the selected models and numbers in parentheses before each model refer to the candidate models before selection (Table S3).

| **Models** | **AICc** | **Delta AICc** | **AICc weight** | **AICc cum. weight** |
| --- | --- | --- | --- | --- |
| **(30) GDD mean; Species; Site; Interactions** | **1622.99** | **0.00** | **1.00** | **1.00** |
| (24) GDD mean; Species; Site | 1653.11 | 30.12 | 0.00 | 1.00 |
| (12) GDD mean; Species | 1667.49 | 44.51 | 0.00 | 1.00 |
| (29) GDD max; Species; Site; Interactions | 1673.58 | 50.59 | 0.00 | 1.00 |
| (23) GDD max; Species; Site | 1695.36 | 72.37 | 0.00 | 1.00 |
| (1) Day of year | 1724.92 | 101.93 | 0.00 | 1.00 |
| (11) GDD max; Species | 1784.42 | 161.44 | 0.00 | 1.00 |
| (18) GDD mean; Site | 1809.84 | 186.86 | 0.00 | 1.00 |
| (6) GDD mean | 1812.91 | 189.92 | 0.00 | 1.00 |
| (17) GDD max; Site | 1850.01 | 227.02 | 0.00 | 1.00 |
| (5) GDD max | 1890.37 | 267.38 | 0.00 | 1.00 |
| (31) Frost prob.; Species; Site; Interactions | 2002.32 | 379.33 | 0.00 | 1.00 |
| (13) Frost prob.; Species | 2060.68 | 437.70 | 0.00 | 1.00 |
| (25) Frost prob.; Species; Site | 2062.66 | 439.67 | 0.00 | 1.00 |
| (7) Frost prob. | 2139.35 | 516.36 | 0.00 | 1.00 |
| (19) Frost prob.; Site | 2141.31 | 518.32 | 0.00 | 1.00 |
| (26) Max daily T °C; Species; Site; Interactions | 2366.40 | 743.41 | 0.00 | 1.00 |
| (20) Max daily T °C; Species; Site | 2422.06 | 799.07 | 0.00 | 1.00 |
| (14) Max daily T °C; Site | 2424.40 | 801.41 | 0.00 | 1.00 |

(Table S8 suite)

| **Models** | **AICc** | **Delta AICc** | **AICc weight** | **AICc cum. weight** |
| --- | --- | --- | --- | --- |
| (8) Max daily T °C; Species | 2434.30 | 811.31 | 0.00 | 1.00 |
| (2) Max daily T °C | 2435.98 | 812.99 | 0.00 | 1.00 |
| (21) Mean daily T °C; Species; Site | 2436.75 | 813.76 | 0.00 | 1.00 |
| (15) Mean daily T °C; Site | 2439.26 | 816.28 | 0.00 | 1.00 |
| (9) Mean daily T °C; Species | 2447.76 | 824.77 | 0.00 | 1.00 |
| (3) Mean daily T °C | 2449.49 | 826.50 | 0.00 | 1.00 |
| (4) Min daily T °C | NA | NA | NA | NA |
| (10) Min daily T °C; Species | NA | NA | NA | NA |
| (16) Min daily T °C; Site | NA | NA | NA | NA |
| (22) Min daily T °C; Species; Site | NA | NA | NA | NA |
| (27) Mean daily T °C; Species; Site; Interactions | NA | NA | NA | NA |
| (28) Min daily T °C; Species; Site; Interactions | NA | NA | NA | NA |

Table S9 Results of the AICc model selection to determine the best combination of air temperature variables for predicting the transition from stage five to stage six of spruce bud phenology, bold indicated the selected models and numbers in parentheses before each model refer to the candidate models before selection (Table S3).

| **Models** | **AICc** | **Delta AICc** | **AICc weight** | **AICc cum. weight** |
| --- | --- | --- | --- | --- |
| **(29) GDD max; Species; Site; Interactions** | **2003.92** | **0.00** | **1.00** | **1.00** |
| (23) GDD max; Species; Site | 2018.47 | 14.55 | 0.00 | 1.00 |
| (30) GDD mean; Species; Site; Interactions | 2023.61 | 19.68 | 0.00 | 1.00 |
| (12) GDD mean; Species | 2024.32 | 20.40 | 0.00 | 1.00 |
| (24) GDD mean; Species; Site | 2026.25 | 22.32 | 0.00 | 1.00 |
| (11) GDD max; Species | 2052.07 | 48.14 | 0.00 | 1.00 |
| (1) Day of year | 2085.67 | 81.75 | 0.00 | 1.00 |
| (17) GDD max; Site | 2116.96 | 113.04 | 0.00 | 1.00 |
| (6) GDD mean | 2123.60 | 119.68 | 0.00 | 1.00 |
| (18) GDD mean; Site | 2125.06 | 121.14 | 0.00 | 1.00 |
| (5) GDD max | 2133.21 | 129.28 | 0.00 | 1.00 |
| (31) Frost prob.; Species; Site; Interactions | 2398.94 | 395.01 | 0.00 | 1.00 |
| (25) Frost prob.; Species; Site | 2488.35 | 484.43 | 0.00 | 1.00 |
| (13) Frost prob.; Species | 2508.16 | 504.24 | 0.00 | 1.00 |
| (19) Frost prob.; Site | 2540.27 | 536.34 | 0.00 | 1.00 |
| (7) Frost prob. | 2558.11 | 554.19 | 0.00 | 1.00 |
| (28) Min daily T °C; Species; Site; Interactions | 3750.38 | 1746.46 | 0.00 | 1.00 |
| (22) Min daily T °C; Species; Site | 3829.88 | 1825.96 | 0.00 | 1.00 |

(Table S9 suite)

| **Models** | **AICc** | **Delta AICc** | **AICc weight** | **AICc cum. weight** |
| --- | --- | --- | --- | --- |
| (10) Min daily T °C; Species | 3836.79 | 1832.86 | 0.00 | 1.00 |
| (16) Min daily T °C; Site | 3851.59 | 1847.67 | 0.00 | 1.00 |
| (27) Mean daily T °C; Species; Site; Interactions | 3852.39 | 1848.47 | 0.00 | 1.00 |
| (4) Min daily T °C | 3854.57 | 1850.65 | 0.00 | 1.00 |
| (9) Mean daily T °C; Species | 3861.40 | 1857.48 | 0.00 | 1.00 |
| (21) Mean daily T °C; Species; Site | 3863.17 | 1859.25 | 0.00 | 1.00 |
| (3) Mean daily T °C | 3876.91 | 1872.99 | 0.00 | 1.00 |
| (15) Mean daily T °C; Site | 3878.89 | 1874.97 | 0.00 | 1.00 |
| (26) Max daily T °C; Species; Site; Interactions | 3923.80 | 1919.88 | 0.00 | 1.00 |
| (8) Max daily T °C; Species | 3955.41 | 1951.48 | 0.00 | 1.00 |
| (20) Max daily T °C; Species; Site | 3957.14 | 1953.22 | 0.00 | 1.00 |
| (2) Max daily T °C | 3973.27 | 1969.35 | 0.00 | 1.00 |
| (14) Max daily T °C; Site | 3975.24 | 1971.32 | 0.00 | 1.00 |

Table S10 Results of the mixed binomial regression model for transition stage zero to two showing the coefficients and standard errors for the best air temperature and photoperiod variables per species and sites; boldface type indicates coefficients that differ from zero.

| **Variable** | **Frost probability** | **Photoperiod (hours)** |
| --- | --- | --- |
|  | **Standardised coefficients (SE)** | **Standardised coefficients (SE)** |
| Intercept: Norway spruce in the temperate forest | 0.79 (0.99) | **1.37 (0.47)** |
| Frost prob. or Photoperiod | **-4.69 (0.20)** | **3.83 (0.19)** |
| White spruce | **4.36 (0.41)** | **4.46 (0.44)** |
| Black spruce | 0.05 (0.32) | 0.11 (0.31) |
| Boreal mixedwood forest | **0.56 (0.28)** | **-2.32 (0.28)** |
| White spruce * Frost prob. or Photoperiod | **-2.86 (0.35)** | **2.92 (0.39)** |
| Black spruce * Frost prob. or Photoperiod | 0.12 (0.20) | -0.19 (0.22) |
| Boreal mixedwood forest * Frost prob. or Photoperiod | **1.66 (0.19)** | **-0.47 (0.22)** |

Table S11 Results of the mixed binomial regression model for transition stage two to three showing the coefficients and standard errors for the best air temperature and photoperiod variables per species and sites; boldface type indicates coefficients that differ from zero.

| **Variable** | **Frost probability** | **Photoperiod (hours)** |
| --- | --- | --- |
|  | **Standardised coefficients (SE)** | **Standardised coefficients (SE)** |
| Intercept: Norway spruce in the temperate forest | **-2.71 (1.65)** | **-2.16 (0.59)** |
| Frost prob. or Photoperiod | **-5.43 (0.34)** | **5.59 (0.49)** |
| White spruce | **4.54 (0.49)** | **4.79 (0.54)** |
| Black spruce | **-1.22 (0.52)** | **-1.61 (0.59)** |
| Boreal mixedwood forest | 0.07 (0.38) | **-3.72 (0.48)** |
| White spruce * Frost prob. or Photoperiod | 0.04 (0.43) | **-2.17 (0.56)** |
| Black spruce * Frost prob. or Photoperiod | **-0.98 (0.56)** | 0.29 (0.67) |
| Boreal mixedwood forest * Frost prob. or Photoperiod | **0.56 (0.39)** | **1.31 (0.49)** |

Table S12 Results of the mixed binomial regression model for transition stage three to four showing the coefficients and standard errors for the best air temperature and photoperiod variables per species and sites; boldface type indicates coefficients that differ from zero.

| **Variable** | **GDD  (° C-days)** | **Photoperiod (hours)** |
| --- | --- | --- |
|  | **Standardised coefficients (SE)** | **Standardised coefficients (SE)** |
| Intercept: Norway spruce in temperate forest | **-1.92 (0.57)** | **-2.74 (0.50)** |
| GDD mean or Photoperiod | **5.77 (0.62)** | **5.83 (0.66)** |
| White spruce | **4.15 (0.52)** | **4.10 (0.56)** |
| Black spruce | -0.42 (0.49) | -0.55 (0.92) |
| Boreal mixedwood forest | -0.28 (0.36) | **-3.05 (0.50)** |
| White spruce * GDD mean or Photoperiod | **1.32 (0.71)** | -0.15 (0.76) |
| Black spruce * GDD mean or Photoperiod | **1.48 (0.78)** | **1.84 (1.02)** |
| Boreal mixedwood forest * GDD mean or Photoperiod | 0.05 (0.58) | -0.36 (0.62) |

Table S13 Results of the mixed binomial regression model for transition stage four to five showing the coefficients and standard errors for the best air temperature and photoperiod variables per species and sites; boldface type indicates coefficients that differ from zero.

| **Variable** | **GDD  (° C-days)** | **Photoperiod (hours)** |
| --- | --- | --- |
|  | **Standardised coefficients (SE)** | **Standardised coefficients (SE)** |
| Intercept: Norway spruce in temperate forest | **-2.74 (0.65)** | **-6.62 (1.09)** |
| GDD mean or Photoperiod | **7.21 (0.93)** | **13.19 (1.40)** |
| White spruce | **4.10 (0.75)** | **5.76 (1.12)** |
| Black spruce | **-4.47 (0.94)** | **-7.87 (1.56)** |
| Boreal mixedwood forest | **-1.47 (0.56)** | **-6.33 (1.00)** |
| White spruce * GDD mean or Photoperiod | **5.31 (0.99)** | -1.07 (1.24) |
| Black spruce * GDD mean or Photoperiod | **2.90 (1.01)** | **6.30 (1.66)** |
| Boreal mixedwood forest * GDD mean or Photoperiod | **-2.29 (0.77)** | **-2.35 (1.00)** |

Table S14 Results of the mixed binomial regression model for transition stage five to six showing the coefficients and standard errors for the best air temperature and photoperiod variables per species and sites; boldface type indicates coefficients that differ from zero.

| **Variable** | **GDD  (° C-days)** | **Photoperiod (hours)** |
| --- | --- | --- |
|  | **Standardised coefficients (SE)** | **Standardised coefficients (SE)** |
| Intercept: Norway spruce in temperate forest | **-2.87 (0.52)** | NA |
| GDD max | **5.98 (0.49)** | NA |
| White spruce | **0.97 (0.60)** | NA |
| Black spruce | **-5.91 (0.94)** | NA |
| Boreal mixedwood forest | **-3.49 (0.68)** | NA |
| White spruce * GDD max | **1.38 (0.58)** | NA |
| Black spruce * GDD max | **2.55 (0.71)** | NA |
| Boreal mixedwood forest * GDD max | **1.01 (0.55)** | NA |

Table S15 Results of the AICc model selection to determine the best combination of photoperiod variables for predicting the transition from stage zero to stage two of spruce bud phenology, bold indicated the selected models and numbers in parentheses before each model refer to the candidate models before selection (Table S4).

| **Models** | **AICc** | **Delta AICc** | **AICc weight** | **AICc cum. weight** |
| --- | --- | --- | --- | --- |
| **(6) Photoperiod; Species; Site; Interactions** | **3607.81** | **0.00** | **1.00** | **1.00** |
| (5) Photoperiod; Species; Site | 3699.05 | 91.23 | 0.00 | 1.00 |
| (3) Photoperiod; Species | 3748.94 | 141.13 | 0.00 | 1.00 |
| (4) Photoperiod; Site | 3758.72 | 150.90 | 0.00 | 1.00 |
| (2) Photoperiod | 3785.84 | 178.03 | 0.00 | 1.00 |
| (1) Day of year | 3861.83 | 254.02 | 0.00 | 1.00 |

Table S16 Results of the AICc model selection to determine the best combination of photoperiod variables for predicting the transition from stage two to stage three of spruce bud phenology, bold indicated the selected models and numbers in parentheses before each model refer to the candidate models before selection (Table S4).

| **Models** | **AICc** | **Delta AICc** | **AICc weight** | **AICc cum. weight** |
| --- | --- | --- | --- | --- |
| **(6) Photoperiod; Species; Site; Interactions** | **2210.37** | **0.00** | **1.00** | **1.00** |
| (5) Photoperiod; Species; Site | 2239.27 | 28.90 | 0.00 | 1.00 |
| (3) Photoperiod; Species | 2302.05 | 91.68 | 0.00 | 1.00 |
| (1) Day of year | 2366.18 | 155.81 | 0.00 | 1.00 |
| (4) Photoperiod; Site | 2386.22 | 175.84 | 0.00 | 1.00 |
| (2) Photoperiod | 2412.99 | 202.62 | 0.00 | 1.00 |

Table S17 Results of the AICc model selection to determine the best combination of photoperiod variables for predicting transition from stage three to stage four of spruce bud phenology, bold indicated the selected models and numbers in parentheses before each model refer to the candidate models before selection (Table S4).

| **Models** | **AICc** | **Delta AICc** | **AICc weight** | **AICc cum. weight** |
| --- | --- | --- | --- | --- |
| **(5) Photoperiod; Species; Site** | **1790.18** | **0.00** | **0.57** | **0.57** |
| **(6) Photoperiod; Species; Site; Interactions** | **1790.77** | **0.59** | **0.43** | **1.00** |
| (3) Photoperiod; Species | 1854.18 | 64.00 | 0.00 | 1.00 |
| (4) Photoperiod; Site | 1895.12 | 104.94 | 0.00 | 1.00 |
| (1) Day of year | 1905.80 | 115.62 | 0.00 | 1.00 |
| (2) Photoperiod | 1917.85 | 127.66 | 0.00 | 1.00 |

Table S18 Results of the AICc model selection to determine the best combination of photoperiod variables for predicting the transition from stage four to stage five of spruce bud phenology, bold indicated the selected models and numbers in parentheses before each model refer to the candidate models before selection (Table S4).

| **Models** | **AICc** | **Delta AICc** | **AICc weight** | **AICc cum. weight** |
| --- | --- | --- | --- | --- |
| **(6) Photoperiod; Species; Site; Interactions** | **1643.40** | **0.00** | **1.00** | **1.00** |
| (5) Photoperiod; Species; Site | 1670.69 | 27.29 | 0.00 | 1.00 |
| (1) Day of year | 1724.92 | 81.52 | 0.00 | 1.00 |
| (4) Photoperiod; Site | 1808.03 | 164.63 | 0.00 | 1.00 |
| (3) Photoperiod; Species | 1839.24 | 195.84 | 0.00 | 1.00 |
| (2) Photoperiod | 1902.71 | 259.31 | 0.00 | 1.00 |

Table S19 Results of the AICc model selection to determine the best combination of photoperiod variables for predicting transition from stage five to stage six of spruce bud phenology, bold indicated the selected models and numbers in parentheses before each model refer to the candidate models before selection (Table S4).

| **Models** | **AICc** | **Delta AICc** | **AICc weight** | **AICc cum. weight** |
| --- | --- | --- | --- | --- |
| **(1) Day of year** | **2085.67** | **0.00** | **1.00** | **1.00** |
| (6) Photoperiod; Species; Site; Interactions | 2322.73 | 237.05 | 0.00 | 1.00 |
| (5) Photoperiod; Species; Site | 2375.84 | 290.16 | 0.00 | 1.00 |
| (4) Photoperiod; Site | 2433.20 | 347.52 | 0.00 | 1.00 |
| (3) Photoperiod; Species | 2569.36 | 483.69 | 0.00 | 1.00 |
| (2) Photoperiod | 2586.51 | 500.83 | 0.00 | 1.00 |

Table S20 Results of the mixed binomial regression model for transition stage zero to two showing the coefficients and standard errors for the best intra-species air temperature and photoperiod variables per seed source and sites for white spruce; boldface type indicates coefficients that differ from zero.

| **Variable** | **Frost probability** | **Photoperiod (hours)** |
| --- | --- | --- |
|  | **Standardised coefficients (SE)** | **Standardised coefficients (SE)** |
| Intercept: White spruce J70 in temperate forest | **3.78 (1.10)** | **6.79 (1.52)** |
| Frost prob. or Photoperiod | **-6.64 (0.67)** | **7.79 (0.80)** |
| White spruce K20 | **2.75 (1.09)** | **6.64 (2.17)** |
| White spruce K24 | **3.01 (1.16)** | **4.92 (1.92)** |
| Boreal mixedwood forest | **2.08 (0.73)** | **-5.23 (0.88)** |
| White spruce K20 * Frost prob. or Photoperiod | **-2.73 (1.33)** | **6.62 (2.26)** |
| White spruce K24 * Frost prob. or Photoperiod | **-2.74 (1.34)** | **4.39 (1.92)** |
| Boreal mixedwood forest * Frost prob. or Photoperiod | **0.89 (0.70)** | **-2.57 (0.83)** |

Table S21 Results of the mixed binomial regression model for transition stage two to three showing the coefficients and standard errors for the best intra-species air temperature and photoperiod variables per seed source and sites for white spruce; boldface type indicates coefficients that differ from zero.

| **Variable** | **Frost probability** | **Photoperiod (hours)** |
| --- | --- | --- |
|  | **Standardised coefficients (SE)** | **Standardised coefficients (SE)** |
| Intercept: White spruce J70 in temperate forest | 0.90 (1.41) | **1.20 (0.44)** |
| Frost prob. or Photoperiod | **-4.55 (0.50)** | **3.07 (0.52)** |
| White spruce K20 | **2.18 (0.58)** | **2.01 (0.66)** |
| White spruce K24 | **1.76 (0.61)** | **2.67 (0.80)** |
| Boreal mixedwood forest | **-0.76 (0.45)** | **-3.56 (0.57)** |
| White spruce K20 * Frost prob. or Photoperiod | **1.46 (0.74)** | -0.65 (0.74) |
| White spruce K24 * Frost prob. or Photoperiod | -0.46 (1.06) | **1.63 (1.02)** |
| Boreal mixedwood forest * Frost prob. or Photoperiod | 0.02 (0.61) | **2.93 (0.77)** |

Table S22 Results of the mixed binomial regression model for transition stage three to four showing the coefficients and standard errors for the best intra-species air temperature and photoperiod variables per seed source and sites for white spruce; boldface type indicates coefficients that differ from zero.

| **Variable** | **GDD (° C-days)** | **Photoperiod (hours)** |
| --- | --- | --- |
|  | **Standardised coefficients (SE)** | **Standardised coefficients (SE)** |
| Intercept: White spruce J70 in temperate forest | **0.83 (0.60)** | 0.02 (0.51) |
| GDD mean or Photoperiod | **4.46 (0.87)** | **5.59 (0.88)** |
| White spruce K20 | **3.48 (0.98)** | **2.29 (0.64)** |
| White spruce K24 | **3.70 (0.97)** | **3.13 (0.69)** |
| Boreal mixedwood forest | 0.23 (0.57) | **-3.00 (0.64)** |
| White spruce K20 * GDD mean or Photoperiod | **4.25 (1.31)** | **2.07 (1.00)** |
| White spruce K24 * GDD mean or Photoperiod | **2.92 (1.37)** | 0.97 (1.05) |
| Boreal mixedwood forest * GDD mean or Photoperiod | 0.93 (1.04) | **1.53 (1.02)** |

Table S23 Results of the mixed binomial regression model for transition stage four to five showing the coefficients and standard errors for the best intra-species air temperature and photoperiod variables per seed source and sites for white spruce; boldface type indicates coefficients that differ from zero.

| **Variable** | **GDD (° C-days)** | **Photoperiod (hours)** |
| --- | --- | --- |
|  | **Standardised coefficients (SE)** | **Standardised coefficients (SE)** |
| Intercept: White spruce J70 in temperate forest | **1.77 (1.64)** | -1.33 (1.68) |
| GDD mean or Photoperiod | **11.41 (1.33)** | **10.11 (1.10)** |
| White spruce K20 | **1.21 (0.59)** | **2.16 (0.72)** |
| White spruce K24 | **2.25 (0.69)** | **1.64 (0.75)** |
| Boreal mixedwood forest | **-3.51 (0.55)** | **-7.73 (0.96)** |
| White spruce K20 * GDD mean or Photoperiod | -1.08 (1.36) | **-1.89 (1.05)** |
| White spruce K24 * GDD mean or Photoperiod | **4.03 (1.88)** | **1.27 (1.24)** |
| Boreal mixedwood forest * GDD mean or Photoperiod | **-1.70 (1.30)** | 0.61 (1.19) |

Table S24 Results of the mixed binomial regression model for transition stage five to six showing the coefficients and standard errors for the best intra-species air temperature and photoperiod variables per seed source and sites for white spruce; boldface type indicates coefficients that differ from zero.

| **Variable** | **GDD (°C days)**  **Standardized coeffiicents (SE)** | **Photoperiod (hours)**  **Standardised coefficients (SE)** |
| --- | --- | --- |
| Intercept: White spruce J70 in temperate forest | **-2.20 (0.43)** | NA |
| GDD max | **5.33 (0.49)** | NA |
| White spruce K20 | **2.03 (0.62)** | NA |
| White spruce K24 | **1.15 (0.67)** | NA |
| Boreal mixedwood forest | **-4.75 (0.77)** | NA |
| White spruce K20 * GDD max | **1.96 (0.90)** | NA |
| White spruce K24 * GDD max | **3.63 (1.19)** | NA |
| Boreal mixedwood forest * GDD max | **3.63 (0.95)** | NA |

Table S25 Results of the mixed binomial regression model for transition stage zero to two showing the coefficients and standard errors for the best intra-species air temperature and photoperiod variables per seed source and sites for Norway spruce; boldface type indicates coefficients that differ from zero.

| **Variable** | **Frost probability** | **Photoperiod (hours)** |
| --- | --- | --- |
|  | **Standardised coefficients (SE)** | **Standardised coefficients (SE)** |
| Intercept: Norway spruce K35 in temperate forest | 0.58 (1.07) | **1.05 (0.62)** |
| Frost prob. or Photoperiod | **-3.78 (0.33)** | **3.38 (0.33)** |
| Norway spruce K39 | 0.27 (0.69) | 0.68 (0.74) |
| Norway spruce K50 | -0.56 (0.65) | -0.23 (0.68) |
| Norway spruce K55 | **1.24 (0.66)** | **1.26 (0.70)** |
| Boreal mixedwood forest | **0.70 (0.56)** | **-2.01 (0.59)** |
| Norway spruce K39 * Frost prob. or Photoperiod | **-0.93 (0.44)** | **1.13 (0.51)** |
| Norway spruce K50 * Frost prob. or Photoperiod | **-0.82 (0.41)** | **1.45 (0.51)** |
| Norway spruce K55 * Frost prob. or Photoperiod | **-1.09 (0.44)** | **0.62 (0.46)** |
| Boreal mixedwood forest * Frost prob. or Photoperiod | **0.66 (0.38)** | **-1.03 (0.39)** |

Table S26 Results of the mixed binomial regression model for transition stage two to three showing the coefficients and standard errors for the best intra-species air temperature and photoperiod variables per seed source and sites for Norway spruce; boldface type indicates coefficients that differ from zero.

| **Variable** | **Frost probability** | **Photoperiod (hours)** |
| --- | --- | --- |
|  | **Standardised coefficients (SE)** | **Standardised coefficients (SE)** |
| Intercept: Norway spruce K35 in temperate forest | **-4.39 (2.01)** | **-2.19 (1.03)** |
| Frost prob. or Photoperiod | **-8.02 (1.18)** | **5.42 (1.01)** |
| Norway spruce K39 | -0.75 (1.20) | -1.03 (1.16) |
| Norway spruce K50 | -0.85 (1.09) | **-1.38 (1.17)** |
| Norway spruce K55 | **2.77 (1.02)** | **1.74 (1.03)** |
| Boreal mixedwood forest | **2.46 (0.87)** | **-3.80 (1.26)** |
| Norway spruce K39 * Frost prob. or Photoperiod | -0.04 (1.29) | 0.64 (1.34) |
| Norway spruce K50 * Frost prob. or Photoperiod | -0.25 (1.20) | **1.52 (1.49)** |
| Norway spruce K55 * Frost prob. or Photoperiod | 1.07 (1.07) | -0.86 (1.19) |
| Boreal mixedwood forest * Frost prob. or Photoperiod | 0.89 (0.89) | **2.20 (1.39)** |

Table S27 Results of the mixed binomial regression model for transition stage three to four showing the coefficients and standard errors for the best intra-species air temperature and photoperiod variables per seed source and sites for Norway spruce; boldface type indicates coefficients that differ from zero.

| **Variable** | **GDD  (° C-days)** | **Photoperiod (hours)** |
| --- | --- | --- |
|  | **Standardised coefficients (SE)** | **Standardised coefficients (SE)** |
| Intercept: Norway spruce K35 in temperate forest | **-2.35 (1.07)** | **-3.95 (0.49)** |
| GDD mean or Photoperiod | **4.12 (1.57)** | **6.54 (0.71)** |
| Norway spruce K39 | -1.09 (1.42) | **-1.81 (0.95)** |
| Norway spruce K50 | 0.49 (1.16) | **1.86 (0.96)** |
| Norway spruce K55 | **3.75 (1.19)** | **3.39 (0.52)** |
| Boreal mixedwood forest | **-1.61 (1.11)** | **-5.62 (0.99)** |
| Norway spruce K39 * GDD mean or Photoperiod | **2.88 (1.94)** | **3.17 (0.00)** |
| Norway spruce K50 * GDD mean or Photoperiod | -1.41 (1.71) | **-3.80 (0.00)** |
| Norway spruce K55 * GDD mean or Photoperiod | **5.04 (2.22)** | **0.06 (0.00)** |
| Boreal mixedwood forest * GDD mean or Photoperiod | **4.91 (2.10)** | **3.15 (0.00)** |

Table S28 Results of the mixed binomial regression model for transition stage four to five showing the coefficients and standard errors for the best intra-species air temperature and photoperiod variables per seed source and sites for Norway spruce; boldface type indicates coefficients that differ from zero.

| **Variable** | **GDD  (° C-days)** | **Photoperiod (hours)** |
| --- | --- | --- |
|  | **Standardised coefficients (SE)** | **Standardised coefficients (SE)** |
| Intercept: Norway spruce K35 in temperate forest | **-3.57 (1.42)** | **-10.59 (3.22)** |
| GDD mean or Photoperiod | **5.99 (1.72)** | **16.03 (4.18)** |
| Norway spruce K39 | **-3.64 (2.34)** | **-6.67 (4.08)** |
| Norway spruce K50 | 1.52 (1.62) | **3.72 (3.07)** |
| Norway spruce K55 | **2.13 (1.45)** | **7.54 (3.22)** |
| Boreal mixedwood forest | -0.80 (1.27) | **-6.87 (3.64)** |
| Norway spruce K39 * GDD mean or Photoperiod | **3.99 (2.67)** | **6.91 (4.62)** |
| Norway spruce K50 * GDD mean or Photoperiod | 0.55 (1.96) | -1.67 (3.41) |
| Norway spruce K55 * GDD mean or Photoperiod | -0.23 (1.95) | **-6.84 (3.65)** |
| Boreal mixedwood forest * GDD mean or Photoperiod | -0.57 (1.59) | -1.36 (3.28) |

Table S29 Results of the mixed binomial regression model for transition stage five to six showing the coefficients and standard errors for the best intra-species air temperature and photoperiod variables per seed source and sites for Norway spruce; boldface type indicates coefficients that differ from zero.

| **Variable** | **GDD  (° C-days)** | **Photoperiod (hours)** |
| --- | --- | --- |
|  | **Standardised coefficients (SE)** | **Standardised coefficients (SE)** |
| Intercept: Norway spruce K35 in temperate forest | **-6.50 (1.60)** | NA |
| GDD max or Photoperiod | **8.71 (1.46)** | NA |
| Norway spruce K39 | -2.21 (2.38) | NA |
| Norway spruce K50 | **2.09 (1.71)** | NA |
| Norway spruce K55 | **6.77 (1.79)** | NA |
| Boreal mixedwood forest | **-3.84 (1.68)** | NA |
| Norway spruce K39 * GDD max or Photoperiod | 1.23 (2.10) | NA |
| Norway spruce K50 * GDD max or Photoperiod | -1.27 (1.47) | NA |
| Norway spruce K55 * GDD max or Photoperiod | **-2.52 (1.57)** | NA |
| Boreal mixedwood forest * GDD max or Photoperiod | 0.12 (1.27) | NA |
